# To Be or Not to Be an Ion Channel: Cryo-EM Structures Have a Say

**DOI:** 10.3390/cells12141870

**Published:** 2023-07-17

**Authors:** Gui-Lan Chen, Jian Li, Jin Zhang, Bo Zeng

**Affiliations:** 1 Key Laboratory of Medical Electrophysiology, Ministry of Education & Medical Electrophysiological Key Laboratory of Sichuan Province, Institute of Cardiovascular Research, Southwest Medical University, Luzhou 646000, China; 2 College of Pharmaceutical Sciences, Gannan Medical University, Ganzhou 341000, China; 3 School of Basic Medical Sciences, Nanchang University, Nanchang 330031, China

**Keywords:** ion channel, cryo-EM, protein structure, transmembrane protein, electrophysiology

## Abstract

Ion channels are the second largest class of drug targets after G protein-coupled receptors. In addition to well-recognized ones like voltage-gated Na/K/Ca channels in the heart and neurons, novel ion channels are continuously discovered in both excitable and non-excitable cells and demonstrated to play important roles in many physiological processes and diseases such as developmental disorders, neurodegenerative diseases, and cancer. However, in the field of ion channel discovery, there are an unignorable number of published studies that are unsolid and misleading. Despite being the gold standard of a functional assay for ion channels, electrophysiological recordings are often accompanied by electrical noise, leak conductance, and background currents of the membrane system. These unwanted signals, if not treated properly, lead to the mischaracterization of proteins with seemingly unusual ion-conducting properties. In the recent ten years, the technical revolution of cryo-electron microscopy (cryo-EM) has greatly advanced our understanding of the structures and gating mechanisms of various ion channels and also raised concerns about the pore-forming ability of some previously identified channel proteins. In this review, we summarize cryo-EM findings on ion channels with molecular identities recognized or disputed in recent ten years and discuss current knowledge of proposed channel proteins awaiting cryo-EM analyses. We also present a classification of ion channels according to their architectures and evolutionary relationships and discuss the possibility and strategy of identifying more ion channels by analyzing structures of transmembrane proteins of unknown function. We propose that cross-validation by electrophysiological and structural analyses should be essentially required for determining molecular identities of novel ion channels.

## 1. Introduction

Ion channels are transmembrane protein complexes with internal pathways for ions to diffuse down their electrochemical gradients rapidly and passively. The opening and closing (gating) of the ion-conducting pore are tightly controlled by factors such as membrane potential (voltage-gated channels), ion concentration (osmosensitive or Ca^2+^/Na^+^/proton-regulated channels), high temperature dependence (thermosensitive channels), mechanical force (mechanosensitive channels) and specific molecules that bind and alter the conformation of the channel (ligand-gated channels). In addition to distinct activation mechanisms, ion channels can also be classified according to their permeant ions, including Na^+^, K^+^, Ca^2+^, Mg^2+^, Cl^−^, H^+^, and others, and those permeable to multiple cations, anions, or both are termed non-selective channels. Ion channels are involved in many critical physiological functions and pathogenesis of diseases not only affecting nervous and cardiovascular systems but also other tissues and organs, including various types of cancer [1,2,3].

So far more than 250 ion channels from ~40 families have been recognized in the human proteome, a huge achievement after the development of the patch clamp [4], molecular biology and biochemical techniques, and the sequencing of genomes. In the 1980s, molecular identification of ion channels largely depended on protein purification from tissue/cell fractions (e.g., acetylcholine receptor [5], voltage-gated sodium channel [6], L-type calcium channel [7], IP_3_ receptor [8], and ryanodine receptor [9]) and positional cloning of genes from patients (e.g., cystic fibrosis transmembrane conductance regulator (CFTR) [10]) and *Drosophila* mutant strains (e.g., Shaker potassium channel [11,12] and transient receptor potential (TRP) channel [13]). When transcript sequence datasets became available, novel proteins with sequence similarity to previously identified ion channels were recognized via homology searches, such as mammalian TRP channels [14,15], the cation channel of sperm (CatSper) [16,17], and voltage-gated proton channel Hv1 [18,19]. In the genomic era, genome-wide RNAi screening of transmembrane proteins has uncovered the molecular identities of store-operated calcium channel (STIM1/Orai1) [20,21,22], volume-regulated anion channel (VRAC) [23,24] and proton-activated chloride (PAC) channel [25]. However, such large-scale fluorescent imaging-based screening is not always necessary as patch clamp or imaging with small-scale pre-selected candidates (mainly according to their enriched expression in specific cell types or localization in organelles) has led to the discovery of mechanosensitive channel Piezo1 [26], the proton channel Otopetrin 1 (Otop1) [27], the lysosomal proton/potassium channel TMEM175 [28,29], the mitochondrial calcium uniporter MICU1/MCU [30,31,32] and the calcium homeostasis modulator 1 (CALHM1) [33].

**Figure 1:**
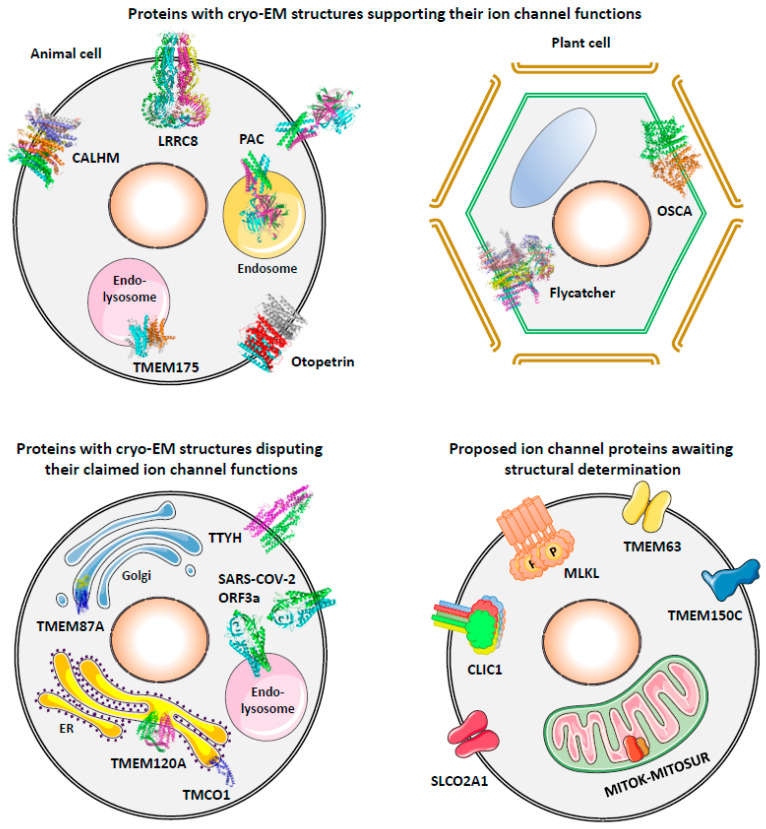
Subcellular localization of proposed ion channel proteins discussed in this review.

In addition to the above successes in molecular identification of ion channels with well-characterized currents, one can imagine that there were numerous attempts that have failed to find proteins conducting these currents, with negative results not published though they are useful for others to exclude ineligible candidates. In contrast, there are also published studies that have misidentified pore-forming proteins of some ion channels [34,35,36,37,38,39]. With the technical advances in cryo-electron microscopy (cryo-EM), protein structures of various ion channels have been resolved and provided substantial knowledge for their working mechanisms since 2013 [40]. On the other hand, cryo-EM structures can also be used to discriminate misidentified channel proteins if they are found unlikely to form an ion-conducting pore. In the following sections, we first present a structure-based classification of mammalian ion channels, and then review cryo-EM evidence that supports or disputes the molecular identities of ion channels identified in recent ten years and discuss the potential validity of proposed ion channels without available protein structures from evolutionary and functional perspectives (Figure 1).

## 2. Structure-Based Classification of Mammalian Ion Channels

Although it is theoretically possible that a protein with multiple TMs may be able to form a functional ion channel in a monomeric architecture, ion channels recognized so far are all in the form of oligomer or monomer with tandemly arranged structurally duplicated domains (Table 1), suggesting that structural symmetry may be essential for the precisely regulated ion-conduction process.

CFTR is a Cl^−^ channel evolved from the ABC transporter family proteins. It keeps the typical structure of ABC transporters which contains two symmetrical transmembrane domains each with 6 TMs [41] and therefore presents a pseudodimeric architecture with a central pore slightly biased toward the N-terminal half [42]. The ClC family of Cl^−^ channels are also dimeric, however, some of them (ClC3-7) are Cl^−^/H^+^ antiporters rather than Cl^−^ channels [43]. Each protomer of ClC-1, ClC-2, hClC-Ka, and hClC-Kb contains 17 or 18 TMs and a pore and dimerizes into “double-barreled” Cl^−^ channels. TMEM16A-H/J/K (ANO1-10), TMEM63A/B/C, and TMC1-8 belong to an unexpected family of dimeric channels with surprising structural similarity [44]. TMEM16 proteins are either Ca^2+^-activated Cl^−^ channels or phospholipid scramblase, while TMEM63 and TMC proteins are mechanosensitive channels. Each protomer of these channels contains 10 or 11 TMs and a potential ion-conducting pathway and assembles into a dimeric complex with 2 putative pores [44].

**Table 1:** Structure-based classification of mammalian ion channels.

Structural Family	Channel	TMs in Each Pore-Forming Protomer	Protomers in Each Channel Complex	Putative Pores in Each Channel Complex	Reference
ABC transporter pseudodimer	Cystic fibrosis transmembrane conductance regulator (CFTR)	2 × 6 TMs	1-mer	1 pore	[42]
Dimer	ClC chloride channels	17/18 TMs	2-mers	2 pores	[45]
TMEM16-like dimer	Calcium-activated chloride channels (TMEM16)	10 TMs	2-mers	2 pores	[46]
	Transmembrane channel-like (TMC)	10 TMs	2-mers	2 pores	[44]
	TMEM63 channels	11 TMs	2-mers	2 pores	
Trimer	Acid-sensing ion channels (ASICs)	2 TMs	3-mers	1 pore	[47]
	Epithelial sodium channel (ENaC)	2 TMs	3-mers	1 pore	[48]
	Proton-activated chloride (PAC) channel	2 TMs	3-mers	1 pore	[49]
	P2X receptors	2 TMs	3-mers	1 pore	[50]
	Trimeric intracellular cation (TRIC) channels	7 TMs	3-mers	1 pore	[51]
	Piezo channels	38 TMs	3-mers	1 pore	[52]
Voltage-gated-like tetramer/ pseudotetramer	Voltage-gated calcium (Ca_V_) channels	4 × 6 TMs	1-mer	1 pore	[53]
	Voltage-gated sodium (Na_V_) channels	4 × 6 TMs	1-mer	1 pore	[54]
	Voltage-gated potassium (K_V_) channels	6 TMs	4-mers	1 pore	[55]
	Calcium-activated potassium (K_Ca_) channels	6 TMs	4-mers	1 pore	[56]
	Sodium-activated potassium (K_Na_) channels	6 TMs	4-mers	1 pore	[57]
	Hyperpolarization-activated cyclic nucleotide–gated (HCN) channels	6 TMs	4-mers	1 pore	[58]
	Cyclic nucleotide-gated (CNG) channels	6 TMs	4-mers	1 pore	[59]
	Cation channel of sperm (CatSper)	6 TMs	4-mers	1 pore	[60]
	Transient Receptor Potential (TRP) channels	6 TMs	4-mers	1 pore	[40]
	Two-Pore channels (TPC)	2 × 6 TMs	2-mers	1 pore	[61]
	Inwardly rectifying potassium (K_ir_) channels	2 TMs	4-mers	1 pore	[62]
	Two-pore domain potassium (K_2P_) channels	2 × 2 TMs	2-mers	1 pore	[63]
	Voltage-gated proton (H_V_1) channel	4 TMs	2-mers	2 pores	[64]
Tetramer/ pseudotetramer	Mitochondrial calcium uniporter (MCU)	2 TMs	4-mers	1 pore	[65]
	Ionotropic glutamate receptors	3 TMs	4-mers	1 pore	[66]
	IP_3_ receptors (IP_3_Rs)	6 TMs	4-mers	1 pore	[67]
	Ryanodine receptors (RyRs)	6 TMs	4-mers	1 pore	[68]
	Lysosomal proton/potassium channel (TMEM175)	2 × 6 TMs	2-mers	1 pore	[69]
	Otopetrin (OTOP) proton channels	2 × 6 TMs	2-mers	4 or 6 pores	[70]
Pentamer	5-HT_3_ receptors	4 TMs	5-mers	1 pore	[71]
	GABA_A_ receptors	4 TMs	5-mers	1 pore	[72]
	Glycine receptors	4 TMs	5-mers	1 pore	[73]
	Nicotinic acetylcholine receptors	4 TMs	5-mers	1 pore	[74]
	Zinc-activated channel (ZAC)	4 TMs	5-mers	1 pore	
Hexamer	Store-operated calcium channels (Orai)	4 TMs	6-mers	1 pore	[75]
Large pore channels (≥6-mers)	Volume regulated anion channel (VRAC/LRRC8)	4 TMs	6-mers	1 pore	[76]
	Gap junctional channels/hemichannels (connexins and pannexins)	4 TMs	6-mers	1 pore	[77]
	Calcium homeostasis modulators (CALHMs)	4 TMs	7–13-mers	1 pore	[78]

Trimeric channels are the family with the most diverse members: except ASIC and ENaC, there is no homology among other members (PAC, P2X, TRIC, and Piezo) and each of them has a unique activation mechanism. Although the number of TMs varies substantially for these channels (2 TMs for ASIC/ENaC/PAC/P2X, 7 TMs for TRIC, and 38 TMs for Piezo), they all possess only a central pore in the trimeric complex [47,48,49,50,51,52].

Voltage-gated-like channels are the largest family of channels with a common evolutionary origin. The majority of them adopt a tetrameric or pseudotetrameric architecture with a central pore formed by TM5 and TM6, including the so-called two-pore channels (TPCs). A protomer of TPCs has two tandem 6-TM regions and thus two pore-forming domains (TM5-6 and TM11-12), which assemble a central pore in a complex consisting of two protomers [61]. The same issue is for the two-pore domain K^+^ (K_2P_) channels, which contain two tandem pore-forming domains (TM1-2 and TM3-4) in each protomer, and mimic the tetrameric architecture of 2-TM inwardly rectifying potassium (K_ir_) channels by dimeric assembly [63]. An exception in the voltage-gated-like family is the voltage-gated proton channel H_v_1, which contains only the voltage sensor domain (TM1-4) and lacks the canonical pore-forming domain (TM5-6). Each H_v_1 protomer has a proton-conducting pathway that opens cooperatively in the dimeric complex [79].

Other tetrameric channels sharing no obvious homology with voltage-gated channels are also quite different from each other, except IP_3_Rs and RyRs which are evolutionarily related to large Ca^2+^ channels at the sarco/endoplasmic reticulum [80]. The two novel proton channels, TMEM175 and Otopetrins, both have two tandem 6-TM regions in each protomer and form a pseudotetrameric channel complex with two protomers. However, TMEM175 has only a central pore [69], whereas Otopetrins may possess four or six pores in each channel complex [70,81]. The remaining two types of tetrameric channels, mitochondrial calcium uniporter (MCU) and ionotropic glutamate receptors have their independent evolutionary origins [65,66].

Pentameric channels, including 5-HT3, GABA_A_, glycine and nicotinic acetylcholine receptors, and zinc-activated channel (ZAC), are all 4-TM ligand-gated channels with a common origin [82]. As the pore-forming subunit of the store-operated Ca^2+^ channel, *Drosophila* Orai adopts a hexameric structure [75], while the structures of mammalian Orai1/2/3 have not been obtained yet. The three types of large pore channels with less selectivity for ions and small molecule metabolites (VRAC, gap junctions, and CALHMs) are structurally similar but with distinct activation mechanisms and electrophysiological properties [83], therefore may have emerged independently during evolution.

## 3. Proteins with Cryo-EM Structures Supporting Their Ion Channel Functions

### 3.1. TMEM175, a Lysosomal Proton and K^+^ Channel

*TMEM175* is a risk gene for Parkinson’s disease first identified by a genome-wide association study in 2014 [84]. It was subsequently found to encode a K^+^ channel in endosomes and lysosomes [28]. Deficiency of TMEM175 disturbs lysosomal membrane potential, luminal pH stability, and autophagosome clearance, resulting in alpha-synuclein aggregation in neurons [28,85]. Two recent studies suggested that TMEM175 is highly permeable to protons at acidic pH with P_H_/P_K_ of ~50,000 or even higher [29,86]. Lack of TMEM175-mediated proton release causes over-acidification of lysosomal lumen which compromises the activities of lysosomal enzymes and leads to alpha-synuclein aggregation in vivo [29]. More independent studies are required to reach an agreement on the proton and K^+^ selectivity of TMEM175.

Although not possessing lysosomes and other organelles, bacteria also have homologs of TMEM175 to form K^+^-permeable channels. Crystal structures of two bacterial TMEM175 homologs showed a tetrameric architecture with six transmembrane helices (TMs) in each protomer (Figure S1A) [87,88]. The putative K^+^-conducting pore is formed by four TM1 helices located at the center of the tetramer, without the TVGYG selectivity filter common for other 6-TM tetrameric K^+^ channels [87]. Human TMEM175 channels have a conserved overall topology similar to its bacterial homologs except that each human protomer contains 12 TMs that can be divided into structurally similar 6-TM N and C domains (Figure S1B) [69]. Therefore, the dimeric assembly of human TMEM175 is actually a pseudotetramer mimicking its bacterial homologs. As the N and C domains of human TMEM175 share ~23% sequence identity, gene duplication and fusion could have occurred during evolution and resulted in the tandem structure of human TMEM175 and its homologs in other vertebrates. The central ion-conducting pore of human TMEM175 is restricted by residues on TM1 and TM7, which share ~40% sequence identity. Consistent with electrophysiological studies, the cryo-EM structure of human TMEM175 obtained at pH 7.4 exhibited an open conformation whereas that at pH 5.5 represented a closed conformation for K^+^ [86]. Molecular dynamics simulation suggests that proton and K^+^ may use the same pathway and the change in their relative permeability at different pH could result from their competition for hydrophilic residues during constriction/dilation of the pore [86]. To better understand the gating mechanism of TMEM175 for proton and K^+^ permeation, it will be of great interest to test the pH sensitivity and proton permeability of bacterial TMEM175 and resolve their structures at acidic pH.

### 3.2. Otopetrin 1 (Otop1), Proton Channel as the Sour Taste Receptor

Early in 2003, mutations in the *Otop1* gene were found to be responsible for the dysfunction of the vestibular system in mutant mouse lines by affecting the formation of otoconia [89], the biomineral particles in the inner ear that transduce gravity and motion to sensory hair cells. The molecular function of Otop1 and its homologs Otop2/3 as proton-selective channels was uncovered in 2018 by comparative transcriptomics of taste receptor cells with and without Zn^2+^-sensitive proton current [27]. Sour taste in mice lacking *Otop1* and *Drosophila* with mutant *Otopetrin-like A* (*OtopLA*) are substantially attenuated [90,91,92], confirming its role as the sour taste receptor.

Cryo-EM structures of zebrafish Otop1, chicken Otop3, and *Xenopus* Otop3 showed conserved dimeric architecture of this channel family, with 12 TMs in each subunit (Figure S2A,B) [70,81]. Interestingly, similar to that observed for TMEM175, each Otop subunit can be divided into two structurally mimicking halves (Figure S2B,C), although there is no homology on their sequences. In each half of the subunit, an α-helical barrel formed by the TMs is observed, which contains a glutamine–asparagine–tyrosine triad at the most constricted position. Mutation of critical residues in either the N- or C-half domain diminished the proton currents, suggesting both of them are probable proton-conducting pores (Figure S2A) [70,81]. Additionally, the intra-subunit interface between the two halves contains functionally important residues conserved in all otopetrins and is likely capable of conducting protons, making it the third potential pathway for proton conduction (Figure S2A) [70]. Unlike TMEM175, the central tunnel between the two Otop subunits is highly hydrophobic and occupied by lipids, which blocked water permeation in molecular dynamics simulation, suggesting this inter-subunit interface is unlikely to form an ion conduction pathway. Since all the structures of Otop1 and Otop3 reported so far are likely in the closed state, further studies resolving the open channel structure are required to clarify the authentic proton-conducting pore in this family of channels with a unique structural arrangement.

### 3.3. Proton-Activated Chloride (PAC) Channel

An acid-sensitive outward-rectifying (ASOR) Cl^−^ current was initially described in rat Sertoli cells in 2003 [93]. A similar current activated by low extracellular pH (<5.5) was then characterized in HEK293 cells in 2005 [94], with permeability to anions in the order of SCN^−^ > I^−^ > NO_3_^−^ > Br^−^ > Cl^−^. This proton-activated anion current was subsequently identified in human epithelial cancer cells (Hela) [95], erythrocytes [96] and epidermoid cancer cells (KB-3-1) [97], and mouse cortical neurons [98] and distal tubular cells (IMCD3) [99]. The molecular identity of this channel was unveiled in 2019 [25,100]. The transmembrane protein TMEM206 was demonstrated to form the channel pore with functional properties consistent with previously reported endogenous ASOR channel, and thus renamed proton-activated chloride (PAC) channel. PAC is ubiquitously expressed in all tissues and cell types, and involved in brain damage after ischemia [25] and regulation of endosomal pH and Cl^−^ level [101].

Cryo-EM studies revealed the trimeric assembly of PAC, with two TMs in each subunit and a ball-shaped extracellular domain (Figure S3) [49]. The structures obtained at pH8 for human and pufferfish PAC represented the closed state, whereas the human PAC structure at pH 4 exhibited a proton-bound non-conducting conformation [49,102]. The open channel structure of human PAC was recently obtained from both detergent and lipid nanodisc samples at pH 4.5, in which a channel pore wider than 3 Å that allows hydrated Cl^−^ to pass through was presented [103]. The distinct structures of PAC at activated, closed, and desensitized states (Figure S3), and the mutagenesis study of key residues that alter pH sensitivity and ion selectivity of the channel have well documented the molecular architecture and gating mechanism of the PAC channel.

### 3.4. Volume-Regulated Anion Channel (VRAC)

Once exposed to a hypotonic environment, cells swell first and then release ions and water to reduce the volume to the normal range. Swelling-activated anion currents were first described in human lymphocytes in 1982 [104]. The molecular identity of VRAC remained elusive until 2014 when LRRC8A (leucine-rich repeat-containing 8 A, or called SWELL1) was identified as an essential component of VRAC [23,24]. LRRC8A alone is found not sufficient to form functional VRAC, instead, it assembles with other LRRC8 proteins (LRRC8B/C/D/E) to form heteromers with variable inactivation kinetics [23]. LRRC8 complex is activated by low ionic strength but not an increase in membrane tension [105], which occur almost concurrently before channel activation. There are also experiments showing that diacylglycerol signaling but not reduced intracellular ionic strength is responsible for VRAC activation [106]. Therefore, the exact gating mechanism of VRAC still needs to be determined by further studies.

Cryo-EM structures of LRRC8 proteins available to date include homomeric LRRC8A [76,107], DCPIB-inhibited LRRC8A [108], and homomeric LRRC8D [109]. All these structures are hexamers consisting of a trimer of dimers for LRRC8A or a dimer of trimers for LRRC8D (Figure S4). The LRRC8 channel contains four structural layers: extracellular domain (ECD), transmembrane domain (TMD), intracellular linker (ICL), and leucine-rich repeats (LRR) (Figure S4A). The putative ion-conduction central pore of LRRC8A is most constricted by Arg103 in the ECD to ~7–8 Å in diameter, which may be the structural basis for anion selectivity [76,107]. The VRAC inhibitor DCPIB binds Arg103 and blocks the extracellular mouth of the LRRC8A channel [108]. Efforts have also been made to resolve the LRRC8A/C heteromeric structure, however, the resolution of which is too low (7.94 Å) to identify the arrangement of LRRC8A/C subunits [76]. Due to the striking functional difference between LRRC8A homomer and native VRAC [110], heteromeric structures of LRRC8A with other LRRC8 members under different ionic strengths are required to elucidate the activation mechanism of VRAC.

### 3.5. Calcium Homeostasis Modulators (CALHMs), Non-Selective Channels with Large Pores

CALHMs are a family of voltage-gated non-selective ion channels inhibited by extracellular calcium [111]. CALHM1 is permeable to both cations and anions, including small-molecule metabolites like ATP, whereas the ion channel activity of other vertebrate CALHMs (CALHM2-6) has not been consistently evidenced [83]. CALHM1 is expressed in the brain and critical for neuronal excitability and calcium homeostasis in response to a decrease in extracellular calcium [112]. Although originally proposed to be a risk locus of Alzheimer’s disease (AD) [33], the association between CALHM1 polymorphism and AD has been challenged by more investigations in different ethnic populations [83,113]. Expression of CALHM1 in taste buds determines ATP release from taste cells that sense sweet, bitter, and umami substances and subsequent purinergic signaling in afferent neurons [114]. Without CALHM1-mediated ATP release, the mice lost much of the three tastes above, but the perception of acid and salt remained unaffected [114]. By far, little is known about the physiological or pathological functions of CALHM1 and its homologs in addition to sense of taste and neurological disorders.

Unlike other channels with fixed numbers of protomers in oligomeric assembly, CALHM members showed quite variable oligomeric compositions (Figure S5A): 7-mer (CALHM1) [115], 8-mer (CALHM1) [115,116,117,118], 9-mer (*C. elegans* CLHM-1) [116], 10-mer (CALHM5/6) [119], 11-mer (CALHM2/5/6) [78,116,117], 12-mer (CALHM5) [120] and 13-mer (CALHM5) [120]. In addition to forming (hemi)channels, CALHMs also assemble into junctions in a tail-to-tail or head-to-head configuration, also with different oligomeric states (Figure S5B): 10-mer junction (CALHM4 and *C. elegans* CLHM-1) [119,121] and 11-mer junction (CALHM2/4) [78,119]. It is unknown whether these variable oligomeric compositions represent their native states or are artifacts resulting from overexpression and high protein density during cryo-EM sample preparation. All these CALHM structures are in open conformations with a pore size large enough to permeate ATP (~14 Å). However, almost no insight into the gating mechanisms of CALHMs has been provided due to the lack of structures with closed conformation. As lipid-like density is observed in the pore of CALHM2/4/5 [117,119,120], further experimentation is required to test the hypothesis that lipids may participate in the regulation of pore size and ion permeation.

### 3.6. OSCA, Osmo/Mechanosensitive Channels in Plants

OSCA is a large family of proteins in plants originally found to be hyperosmolarity-activated Ca^2+^-permeable channels in *Arabidopsis* by mutant screening and expression cloning in 2014 [122,123]. With this osmosensor, plants can regulate transpiration and growth to adapt to water deficiency and osmotic stress. The mechanosensitive nature of OSCA channels was unveiled by two independent groups in 2018 [124,125]. Heterologous expression of various OSCA proteins produced robust membrane stretch-activated currents in PIEZO1-KO HEK293 cells, and the mechanosensitivity and pore-forming role of OSCA1.2 was further confirmed by reconstitution of proteins in liposomes [124].

Cryo-EM structures of OSCA1.1, OSCA1.2, and OSCA3.1 share a surprisingly similar dimeric architecture with TMEM16 proteins (Figure S6), a family including Ca^2+^-activated ion channels and phospholipid scramblases [125,126,127,128]. Each OSCA subunit contains 11 TMs, with the latter 10 TMs well matching the 10 TMs in TMEM16 proteins. Although the structures of OSCA proteins obtained are all in a closed conformation, a putative ion conduction pore could still be identified in each subunit (Figure S6A). The pores of OSCA proteins resemble the shape of an hourglass and are constricted by a series of hydrophobic residues located in different TMs, and are clearly dilated by surface tension in molecular dynamics simulation [125]. A notable difference between OSCA and TMEM16 proteins is that OSCA structures do not possess a stable Ca^2+^-binding site in the region corresponding to the negatively charged residues-rich domain of TMEM16A [125,126,127,128]. Several modes of mechanosensitive gating of OSCA channels have been proposed based on molecular dynamics simulations, including the movement of TMs and stretch of the long helices and linkers in the intracellular domain [125,126,127,128]. However, solid evidence can only come from structures showing the open channel conformation, which may be a great challenge for mechanically gated channels without small molecule agonists available.

### 3.7. Flycatcher1, the Touch-Sensitive Channel of Venus Flytrap

Flycatcher1 is a stretch-activated channel identified in the sensory hair on the leaf of the Venus flytrap [129], a well-known carnivorous plant (Figure S7A). Flycatcher1 belongs to the family of bacterial mechanosensitive channels of small conductance (MscS)-like (MSL) ion channels in plants. Heterogenous expression of Flycatcher1 in PIEZO1-KO HEK cells results in large stretch-activated Cl^−^ currents [129]. The efflux of Cl^−^ from sensory cells of the Venus flytrap upon mechanical stimulation would induce membrane depolarization [129] and subsequent Ca^2+^ influx and propagation in the leaf, which triggers trap closure [125]. Interestingly, homologs of Flycatcher1 are also highly expressed in the touch-sensitive structures of Cape sundew, another carnivorous plant in the family of Venus flytrap but with distinct morphology (Figure S7B) [129]. Stretch-activated Cl^−^ currents have also been recorded from the mechanosensitive cells in the leaflet of *Mimosa pudica*, a plant well known for its rapid leaflet movement in response to touch (Figure S7C) [130], implying that homolog of Flycatcher1 may also exist and function as the mechanosensor in this distally related species.

The cryo-EM structure of Flycatcher1 revealed a heptameric architecture (Figure S7D) resembling that of the bacterial MscS and plant MSL1 channels, with both N and C termini intracellularly located [131]. Each Flycatcher1 subunit contains six TMs, of which TM1/2/3 are additional to MscS/MSL1. Single-channel conductance and Cl^−^ permeability are affected by lysine residue mutations in the side portals of the cytoplasmic cage. A unique feature of Flycatcher1 is its flexible cytoplasmic TM4-TM5 linker that is much longer than the corresponding region in MscS/MSL1 and exhibits either up or down orientation. This linker interacts with the cytoplasmic cage and its conformational change can modulate channel gating, at least for deactivation kinetics [131].

## 4. Proteins with Cryo-EM Structures Disputing Their Claimed Ion Channel Functions

### 4.1. TMCO1, ER Ca^2+^-Leak Channel or Accessory Subunit of the ER Translocon?

Homozygous frameshift mutation in the *transmembrane and coiled-coil domain-containing protein 1* (*TMCO1*) gene was identified in 11 patients from the Amish community and responsible for their congenital anomalies including craniofacial dysmorphism, skeletal malformation, and intellectual disability [132]. This “TMCO1-defect syndrome” was then found to share many clinical features with cerebrofaciothoracic dysplasia (CFTD) when more pathogenic variants were characterized from other populations [122,133,134]. TMCO1 is a transmembrane protein at the ER and its deficiency resulted in supernormal ER Ca^2+^ level and was therefore proposed to form a Ca^2+^ channel that releases Ca^2+^ when the ER Ca^2+^ store is overfilled [34]. TMCO1 was proposed to assemble an ion-conducting pore in a tetrameric architecture, and purified TMCO1 proteins were capable to form functional Ca^2+^-selective channels in reconstituted proteoliposomes [34].

Nevertheless, a cryo-EM study that isolated TMCO1-ribosome complexes with Flag-tagged TMCO1 as a bait demonstrated that TMCO1 is associated with the Sec61 ER translocon as an accessory component [135]. TMCO1 is present in the macrocomplex as a monomer and apparently unable to form a pore by itself (Figure S8). Instead, defect in TMCO1 could affect the biogenesis of a large variety of multi-pass membrane proteins [135], which may include the putative Ca^2+^ load-activated Ca^2+^-leak channel, and cause the abnormal ER Ca^2+^ content in TMCO1-deficient cells. However, a more feasible explanation for the phenotype of TMCO1 deficiency is that the Sec61 complex is not only a pathway for importing polypeptides into the ER but also a Ca^2+^-permeable channel due to its large pore size [136]. Silencing or inhibition of Sec61 suppressed Ca^2+^ leakage from the ER [137,138], while a bacterial exotoxin can facilitate the Ca^2+^ leakage by arresting Sec61 in a Ca^2+^-permeable state [139]. As a critical component of the Sec61 complex, TMCO1 is eligible to function as an auxiliary subunit of the Sec61 channel and modulate its Ca^2+^ permeability in parallel with polypeptide transportation.

### 4.2. TMEM120A, Mechanosensitive Channel or Enzyme in Fat Metabolism?

TMEM120A and TMEM120B are initially found to be transmembrane proteins at the nuclear envelope involved in the differentiation of adipocytes in 2015 [140]. Since 2020, much attention has been drawn to the study showing the role of TMEM120A as a mechanosensitive channel in dorsal root ganglia (DRG) neurons to sense mechanical pain [36]. The new name TACAN (“movement” in Farsi) was suggested for TMEM120A in the same study. Cryo-EM structures of TMEM120A and TMEM120B were soon resolved by several research groups. The structures of TMEM120A from five independent studies, as well as a structure of TMEM120B, are almost the same: symmetrical homodimer with N-terminal coiled-coil domain and C-terminal transmembrane domain with six TMs in each subunit (Figure S9A) [37,38,141,142,143]. The TMEM120A structures are highly similar to that of fatty acid elongase 7 (ELOVL7) (Figure S9B) and contain a coenzyme A molecule bound within the transmembrane domain (Figure S9A). These results suggest that TMEM120A may participate in fatty acid metabolism as an enzyme with unknown catalytic activity.

Moreover, four of these five studies failed to record mechanosensitive currents in cells with TMEM120A overexpression [37,38,142,143]. The remaining one performed a pressure clamp on TMEM120A-transfected COS-7 cells and identified the M207A mutant that generated much larger stretch-activated currents than wildtype TMEM120A [141]. As the authors did not investigate the localization of TMEM120A in COS-7 cells, it is unclear whether the large mechanosensitive currents are due to the channel activity of TMEM120A itself, or its regulation of an endogenous mechanosensitive channel. TMEM120A has been shown to inhibit PIEZO2, but has no effect on PIEZO1 and TREK1 channels [144]. Single-cell RNA sequencing of mechanosensory DRG neurons revealed that TMEM120A is expressed in all subtypes of mechanosensory neurons but dispensable for the mechanosensitive activity in these neurons [145]. In another aspect, adipocyte-specific deletion of TMEM120A in mice results in latent lipodystrophy [146], which is in line with the original finding of the same research group in 2015 [140]. Interestingly, a recent study uncovered the antiviral function of TMEM120A, which is achieved by interacting with STING and promoting STING translocation from the endoplasmic reticulum (ER) to the ER-Golgi intermediate compartment [147]. The above studies suggest that the exact molecular function of TMEM120A in lipid metabolism needs to be recognized to understand its regulatory roles in fat accumulation, mechanosensitive response, and STING activation, though the possibility that an ion-conducting pore exists in the TMEM120A structure cannot be completely excluded [141].

### 4.3. TMEM87A, Mechanosensitive Channel Component or Trafficking Chaperone?

TMEM87A and its homolog TMEM87B are initially recognized as membrane proteins involved in endosome-to-trans-Golgi network (TGN) retrograde transport [148]. Later in 2020, a study found that the mechanically activated currents in melanoma cells are associated with TMEM87A, and expression of TMEM87A induced robust mechanosensitive currents in PIEZO1-KO HEK293 cells [149]. It was thus renamed Elkin1, from the Greek word Elko (“to pull”). The cryo-EM structure of human TMEM87A published recently [150] shows that TMEM87A is a monomer with a sandwich-like extracellular domain and a transmembrane domain of seven TMs (Figure S10). Ion-conducting pores cannot be identified from the structure, and the authors also failed to record mechanosensitive currents from TMEM87A reconstituted proteoliposomes [150]. Further structural analyses revealed that TMEM87A is not a G protein-coupled receptor but shares similarities with several Golgi dynamics (GOLD) domain seven-transmembrane helix (GOST) proteins that function in membrane-associated protein trafficking. Therefore, if a mechanosensitive channel truly exists in melanoma cells, TMEM87A may promote its trafficking to the plasma membrane though it is not specifically involved in this mechano-transduction pathway.

### 4.4. TTYH1/2/3, Another VRAC or Not?

*Tweety* is a gene identified from the *flightless* locus of *Drosophila melanogaster* and hence named after a flightless cartoon bird. Human homologs of *tweety* (TTYH1/2/3) have been proposed to be maxi-Cl^−^ channels or VRAC [151,152,153]. Two recent studies have presented cryo-EM structures of human TTYH1/2/3 and mouse TTYH2/3 with identical dimeric architecture and an extended extracellular domain (Figure S11A) [154,155]. A potential Ca^2+^-binding site is found in the extracellular domain of TTYH2, which may help to stabilize the dimeric association of protomers with negatively charged surfaces [154]. In the absence of Ca^2+^, TTYH2 proteins are either monomers or head-to-head dimers in lipid nanodiscs (Figure S11B,C) [154]. Both studies have failed to find the structural basis of an ion-conducting pore in the TTYH structures. First, TM2-TM5 are tightly packed, and it is unlikely that a minor conformational change could create an aqueous pathway for ions to go through in the hydrophobic microenvironment. Second, the inter-subunit interface is predominantly hydrophobic and incapable of forming a pore. Third, no hydrophilic groove is found in the transmembrane domain that can potentially serve as an ion-conducting path. Fourth, all the residues previously proposed critical for ion conduction are located distally from the membrane and are not useful to indicate the ion-permeation route. In consistency with these structural observations, electrophysiological recordings confirmed that TTYH family proteins do not form ion channels [154,155]. Interestingly, a more recent study using cross-linking and single-molecule fluorescence microscopy demonstrated that TTYH1/3 assembles as tetramers at the plasma membrane, while detergent disrupts tetramers into dimers [156]. This raised the question of whether the cryo-EM structures obtained from purified proteins can represent their native architecture in the cell. Nevertheless, this result also provides evidence that the TTYH complex is not as stable as *bona fide* ion channels.

### 4.5. SARS-CoV-2 Viroporins

Viroporins are viral transmembrane proteins that assemble into oligomeric pores in the plasma membrane or organellar membrane of host cells to allow flux of ions or small molecules [157]. Among the 29 proteins encoded in the SARS-CoV-2 genome, the envelope (E) protein, ORF3a, ORF7b, and ORF10 have been proposed to be ion channels and potential drug targets against viral infection [158,159]. In the meantime, there are also publications criticizing that the currents of these viroporins are more likely technical artifacts or endogenous currents of the expression system [160,161].

To date, the only full-length protein structure of SARS-CoV-2 viroporins is from ORF3a [162]. It forms a dimeric architecture with three TMs in each subunit and C-termini in the cytosol (Figure S12A). A large polar cavity is found in the inner half of the transmembrane domain and opens to the cytosol by water-accessible lower tunnels (Figure S12A). Toward the extracellular or luminal side, hydrophobic residues form a tight seal above the cavity, making it almost impossible to open a central pore for ions to pass through. Instead, the authors suggest that the partially hydrophilic grooves between TM2 and TM3 would be a path for ions, however dramatic conformational change in the outer half of the transmembrane domain is still required. The authors also found that two ORF3a dimers can further assemble into a tetramer by a side-by-side connection between the cytosolic domains, but this contact is limited to eight residues from each side and thus unable to create a pore [162]. Although the existence of an ion-conducting pore in the ORF3a structure is somehow ambiguous, the authors provided evidence for ORF3a being a Ca^2+^-permeable non-selective cation channel by electrophysiological recording and fluorescent imaging in proteoliposomes containing ORF3a and mutants [162].

Most recently, a new study presented cryo-EM structures of ORF3a from SARS-CoV-2 and SARS-CoV-1 [39]. Both structures are dimeric and similar to the previously reported one of SARS-CoV-2. The authors argued that the cavity in the transmembrane domain of ORF3a is surrounded by several positively charged residues that repel cations (Figure S12B), and thus unlikely to form a pore for cation permeation, regardless of whether the captured structures are in a closed state or not. The authors also performed comprehensive electrophysiological experiments including whole-cell and endolysosomal patch clamp in HEK293 cells, and recordings in *Xenopus* oocytes and reconstituted proteoliposomes, but none of these attempts have obtained evidence supporting the channel activity of ORF3a. The large currents from reconstituted proteoliposomes generally result from membrane leakiness and/or contamination of endogenous channel proteins in the purified sample [39].

Different from ORF3a, other proposed SARS-CoV-2 viroporins, including protein E, ORF7b, and ORF10, possess only one or no transmembrane segment (Figure S12C). As currently recognized pore-forming proteins from bacteria to mammals all possess at least 2 TMs, the ability of single-TM proteins to constitute a functional channel would be highly suspicious. The NMR structure of the transmembrane domain of protein E showed a pentameric architecture [163]; however, it is unknown whether the full-length protein E could assemble in the same way. Residues lining the putative central pore of the pentameric complex are predominantly hydrophobic and the structural basis of its claimed cation selectivity is unclear, which would indicate a preference for lipid occupation other than ion permeation. A similar amino acid composition is present in the transmembrane segment of ORF7b (Figure S12C). For ORF10, it should be noted that it is only a hypothetical gene, and its transcript was not detected in the subgenomic RNAs (RNA intermediates for ORF translation, transcribed from viral genomic RNA) of SARS-CoV-2 [164]. The putative ORF10 coding region is intact in only a few cases of coronaviruses closely related to SARS-CoV-2. The pathogenicity and transmissibility of SARS-CoV-2 variants with a premature stop codon in ORF10 were not attenuated, suggesting that ORF10 is not essential for the virus, or it does not exist at the protein level [165].

## 5. Proposed Ion Channel Proteins Awaiting Structural Determination

### 5.1. TMEM63 Proteins as Osmo/Mechanosensitive Channels

TMEM63 proteins are homologs of plant OSCA channels in animals and are proposed to form either osmosensitive [136] or mechanosensitive channels [124]. Three members of the TMEM63 family are present in mammals (TMEM63A/B/C) and only one exists in *Drosophila* (TMEM63). TMEM63A and TMEM63B are co-expressed in most (if not all) tissues and organs in humans and mice, while TMEM63C is mainly expressed in the nervous and endocrine systems. Patients carrying heterozygous missense variants of *TMEM63A* manifested infantile-onset neurodevelopmental disorders due to transient or severe hypomyelination [132,134,166]. Missense variants of *TMEM63B* were identified in patients with intellectual disability and abnormal motor function and brain morphology [167]. No homozygous mutations for *TMEM63A* or *TMEM63B* in humans have been recorded yet. Biallelic variants of *TMEM63C* are found to cause hereditary spastic paraplegias, with mild intellectual impairment in some patients.

Phenotyping data from the International Mouse Phenotyping Consortium (IMPC) showed that constitutive KO of *Tmem63a* resulted in abnormal gait, whereas *Tmem63b*-null mice all died before weaning, and the heterozygotes showed behavioral phenotypes similar to the patients. *Tmem63c*-deficient mice are generally normal and only exhibited morphological changes in the brain, heart, and liver at the late adult stage. A recent study found that TMEM63A is expressed in nociceptors in the dorsal root ganglion of mice and involved in mechanical allodynia [34]. For TMEM63B, the only physiological function reported so far is its role as a hypo-osmolarity-activated channel regulating the survival of cochlear hair cells and which supports hearing in mice [136]. Downregulation of TMEM63C in podocytes is associated with kidney damage in rats and affects glomerular function in zebrafish [168]. In *Drosophila*, TMEM63 is used as a mechanosensor to detect the grittiness of food [131] and environmental humidity [169].

The major controversy on TMEM63 channels is their activation mechanisms in heterologous expression systems, while activation by hyper-osmolarity, hypo-osmolarity, and mechanical stretch have all been reported but cannot be cross-validated [123,124,136,138]. According to AlphaFold prediction, TMEM63 proteins share the same structural arrangement with the OSCA proteins, with 11 TMs in each subunit which place the N- and C-terminus outside and inside the cell, respectively (Figure S13). It can be expected that TMEM63 channels also adopt a dimeric architecture as OSCA channels, however, a detailed comparison between the cryo-EM structures of these two close homologs will help to further understand the structural basis of their ion-conducting pathway, ion selectivity, and gating mechanisms.

### 5.2. TMEM150C/Tentonin3, Mechanosensitive Channel or Just a Regulator?

TMEM150C was found to be a component of a mechanosensitive channel with slow-inactivating kinetics in 2016 and the name Tentonin3 (from the Greek word “Tentono” which means “to stretch”) was proposed by the authors [170]. The deficiency of TMEM150C attenuated the slow adapting mechanically induced currents in DRG neurons and affected motor coordination in mice [170]. Studies from the same research group further found that TMEM150C also participates in the sensing of blood pressure in the aortic arch by functioning as a component of the baroreceptor [171], and contributes to glucose-stimulated insulin secretion in pancreatic β-cells [172]. However, the mechanosensitivity of TMEM150C was argued by the slow inactivating mechanosensitive current was only observed in TMEM150C-transfected HEK293 cells with endogenous expression of Piezo1. In Piezo1-knockout HEK293 (HEK-P1KO) cells, overexpression of TMEM150C did not produce any current that responds to mechanical stimulus [173]. In response to this argument, evidence that partially supports the channel-forming role of TMEM150C was provided by authors of the original article: (1) the currents of Piezo1 and TMEM150C were additive to each other when they were overexpressed in HEK293T cells separately or together; (2) mutations in the putative pore region of TMEM150C altered the relative permeability of Cl^−^ to Na^+^ [174]. Nonetheless, a later study from another independent research group demonstrated that TMEM150C was able to slow down the inactivation of mechanosensitive currents of Piezo1/2 and the two-pore K^+^ channel TREK-1, and overexpression of TMEM150C alone in HEK-P1KO showed no mechanical response [175]. Moreover, a recent study found that TMEM150C also failed to generate a mechanosensitive current in Piezo1-knockout N2A neuroblastoma cells, and the *Tmem150c*^−/−^ mice showed no changes in the activity of cutaneous sensory neurons and motor coordination [176], which further doubted the role of TMEM150C in mechanosensation.

TMEM150C belongs to a transmembrane protein family with two other members TMEM150A and TMEM150B, and all share a highly conserved 6-TM structure according to AlphaFold prediction (Figure S14). As reported, the amplitudes of mechanosensitive currents by TMEM150A/B in HEK293T cells were much smaller than TMEM150C and not significantly different from the GFP-transfected control [170], suggesting the mechanosensitivity of TMEM150A/B should be very weak or none. TMEM150A has been found to interact with phosphatidylinositol 4-kinase type IIIα (PI4KIIIα), the major enzyme responsible for the synthesis of phosphatidylinositol 4-phosphate (PI4P) at the plasma membrane [169]. PI4P is the precursor of phosphatidylinositol 4,5-bisphosphate (PIP_2_), which is a critical phospholipid regulating a variety of ion channels by itself and its downstream metabolites diacylglycerol (DAG) and inositol 1,4,5-trisphosphate (IP_3_) [177]. Overexpression of TMEM150A enhances the rate of PIP_2_ recovery following PIP_2_ depletion by phospholipase C [167,169], implying that it may modulate the activity of ion channels via lipid regulation. Although unable to directly interact with PI4KIIIα, TMEM150B has been shown to perform a similar function as TMEM150A when the C terminal tail of TMEM150A was fused to its full-length sequence [169]. Since no cellular functions of TMEM150C other than its role in mechanosensation have been proposed, it is attractive to investigate whether it has a similar action in phospholipid metabolism as TMEM150A. To better understand the molecular functions of the TMEM150 protein family, cryo-EM structural study will greatly help to determine whether they are pore-forming proteins, facilitators of phospholipid synthesis, or something else.

### 5.3. MITOK and MITOSUR as the Mitochondrial ATP-Sensitive K^+^ Channel

ATP-sensitive K^+^ (K_ATP_) currents across the mitochondrial inner membrane were first described in mitoplasts from rat liver in 1991 [178]. Unlike the plasma membrane K_ATP_ channel (first reported in 1983 [179]) with the sulfonylurea receptor (SUR) and Kir6.2 subunits characterized in 1995 [180,181], the molecular identity of mitoK_ATP_ has remained elusive until 2019. Screening of mitochondrial membrane proteins with unknown functions identified MITOK (encoded by the *CCDC51* gene) and MITOSUR (ATP-binding cassette protein 8, ABCB8) as the pore-forming and regulatory subunits of mitoK_ATP_, respectively [182]. Reconstitution of MITOK and MITOSUR into liposomes produced currents with major properties of mitoK_ATP_. Because MITOK/MITOSUR share no homology in sequences with their counterparts Kir6.2/SUR in the plasma membrane K_ATP_ channel, it is currently unknown whether they could form a mitoK_ATP_ channel in a similar architecture: a pore-forming Kir6.2 tetramer with four peripheral SUR subunits each docked on a Kir6.2 protomer (Figure S15A) [183]. The structure of MITOK (CCDC51) predicted by AlphaFold contains several unordered TMs and is not very informative (Figure S15C). The cryo-EM structure of MITOSUR (ABCB8) exhibited a dimeric architecture typical of ABC transporters [184], which lacks the additional transmembrane domain 0 (TMD0) of SUR subunit that interacts with Kir6.2 (Figure S15D,E). It can be expected that the structure of an MITOK/MITOSUR complex will be presented in the near future to demonstrate whether they are a *bona fide* structural basis of mitoK_ATP_.

### 5.4. SLCO2A1 as the Maxi Cl^−^ Channel

Maxi Cl^−^ channel is an anion-selective channel with large conductance that exists in species from amphibians to mammals [185]. Its activity was initially detected in skeletal muscle cells [186] and subsequently observed in neurons, glia, lymphocytes, cardiomyocytes, and many other cell types [187]. Maxi Cl^−^ channel is considered an important ATP release pathway in addition to gap junctional hemichannels and CALHMs [185]. Attempts to characterize the molecular identity of maxi Cl^−^ channel have excluded a long list of known anion channels, solute carrier (SLC) family transporters, and other transmembrane proteins, and eventually identified SLCO2A1 (Figure S16), a well-recognized prostaglandin transporter [188,189], as the core component of the maxi Cl^−^ channel [190]. When heterologously expressed in cells and reconstituted into liposomes, SLCO2A1 recapitulated the properties of the maxi Cl^−^ channel, and its role in ATP release was also confirmed [190]. Although it is proposed that SLCO2A1 may function as a prostaglandin transporter at resting state, and becomes permeable to Cl^−^ and ATP in the activated state [185], no substantial evidence has been provided for this hypothesis. Cryo-EM study on SLCO2A1 and its potential regulatory proteins ANXA2 and S100A10 [191] will be the most straightforward way to determine whether it is eligible to implement dual functions of prostaglandin transport and anion permeation. The role of SLCO2A1 as the pore-forming subunit of the maxi Cl^−^ channel would be ultimately confirmed only if a large pore lined with positively charged residues is observed.

### 5.5. Mixed Lineage Kinase Domain-like (MLKL) Protein as a Necroptotic Cation Channel

MLKL is the “executioner” of necroptosis [192]. After necroptotic induction and formation of receptor-interacting protein kinase 1 (RIPK1)/RIPK3 complex (necrosome), MLKL is recruited into the necrosome and phosphorylated by RIPK3 [193]. Phosphorylated MLKL is then dissociated from the necrosome, oligomerized, and translocated to the plasma membrane [194]. Aggregation of MLKL oligomers at the plasma membrane permeabilizes the cell and results in lytic cell death (Figure S17A) [195]. Structurally, MLKL possesses an N-terminal transmembrane domain with four TMs and a C-terminal pseudokinase domain interacting with RIPK3 [196]. Full-length MLKL or its N-terminal domain is sufficient to form a cation channel in the planar lipid bilayer, with permeability to Na^+^, K^+^, and Mg^2+^, but not Ca^2+^ [197]. MLKL is found to form tetramers or octamers (dimer of tetramers) before or during translocation [198]; however, there is also a study showing that MLKL forms large amyloid-like polymers with a molecular weight of over 2 million Daltons [199]. The only protein structure of full-length MLKL available so far is the crystal structure of mouse MLKL at an unphosphorylated state, which is simply monomeric (Figure S17B) [196]. The structural basis of the MLKL channel and its ion selectivity is unclear. Cryo-EM study on phosphorylated MLKL oligomers will help to address this issue.

### 5.6. Chloride Intracellular Channels (CLICs), Transformers of Soluble Proteins?

CLICs are a family of evolutionarily conserved proteins in animals, normally with six members (CLIC1-6) in vertebrates [200]. The first CLIC protein (p64) was identified in a study searching for a Cl^−^ channel in the bovine kidney [201,202] and later named CLIC5B. Although sequence analyses of p64 and later discovered NCC27 (CLIC1) and p64H1 (CLIC4) did not show signs of transmembrane proteins, ion channel activities were recorded in microsomes or cells overexpressing these proteins [203,204,205]. The pore-forming properties of CLIC proteins were demonstrated by incorporating purified proteins into artificial lipid bilayers and electrophysiological measurements (reviewed by Littler, Harrop, Goodchild, Phang, Mynott, Jiang, Valenzuela, Mazzanti, Brown, Breit and Curmi [200]).

As the best studied CLIC protein, CLIC1 is a soluble monomeric protein with the typical structure of the glutathione S-transferase (GST) superfamily proteins under reducing condition [206]. Upon oxidation, CLIC1 proteins assemble into non-covalent dimers due to structural rearrangement of the N-terminal domain, in which a disulfide bond (Cys24-Cys59) is formed in each protomer and a large hydrophobic surface is exposed to serve as the dimer interface (Figure S18) [207]. The major structural transition during oxidation is the conversion of the four-stranded β sheet into a helical structure, which is potentially integrated into lipid bilayers as a transmembrane segment [208]. The membrane-spanning CLIC1 proteins then oligomerize into a channel complex (Figure S18) [209]. Nonetheless, it was also shown that soluble CLIC1 proteins can spontaneously insert into the membrane without oxidation [210]. A recent study found that binding of intracellularly released Zn^2+^ triggers CLIC1 insertion into the membrane and Cl^−^ efflux through presumed CLIC1 channels is activated at low pH [211].

In addition to the channel function, CLIC proteins are also found to interact with ezrin, radixin, and moesin (ERM) proteins that crosslink membrane and cortical actin. CLIC and ERM proteins regulate actin-facilitated membrane processes such as microvillar maintenance, phagocytosis, and vesicle trafficking under the control of Rho GTPases (reviewed by Jiang, et al. [212]). It is not surprising that a metamorphic protein can perform distinct functions with different conformations. However, before adding CLICs to the expanding list of metamorphic proteins, it is essential to resolve the structures of all the presumed conformations. If the membrane integral form of CLIC proteins does stably exist, they could be captured with nanodiscs and subject to cryo-EM analysis. The claim that CLICs are an exceptional class of ion channels will be accepted undoubtedly only if a structure of the membrane-spanning complex with internal hydrophilic pathways is observed.

## 6. Perspective: Structure-Guided Discovery of Unrecognized Ion Channels

It has been much more challenging to identify novel ion channels nowadays as most of the characteristic membrane currents have been assigned to ion channels with unambiguous molecular identities. However, there are still a considerable number of transmembrane proteins of unknown function in the human proteome, which potentially contain some pore-forming proteins not yet recognized. Based on different algorithms, the number of transmembrane proteins in the human proteome ranges from 5508 to 7651, ~45% of which are single-TM proteins [213,214]. To assemble an ion channel with transmembrane proteins, amino acid residues lining the TMs should fulfill the following criteria: (1) be overall hydrophobic to ensure stable integration into the membrane; (2) be able to create a hydrophilic pathway for ions to go through; (3) interact with residues in adjacent TMs to stabilize the architecture of the pore. It is almost impossible to allocate all these properties to a single TM, so ion channels recognized so far are all formed by proteins with at least two TMs.

Among the ~3000 (conservative estimate) proteins with two or more TMs, the largest family is the 7-TM G protein-coupled receptors (GPCRs) with more than 800 members [215]. The promiscuous TMEM proteins, which were randomly assigned with numerical names (TMEM1-TMEM275) and sometimes mistakenly called a “family”, represent the most mysterious collection of transmembrane proteins. Some TMEM proteins, including TMEM38A/B (TRIC-A/B), TMEM175, TMEM206 (PAC), and TMEM63A/B/C, have been recognized as ion channels, while some others are found to conduct other cellular functions, such as TMEM30A/B being auxiliary subunits of phospholipid flippases [216]. However, most of the TMEM proteins have not been functionally characterized and are potentially an important source of unidentified ion channels.

The cases of SLCO2A1 and CFTR indicate that the over 400 SLC transporters [217] and 49 ATP-binding cassette (ABC) transporters [218] may also contain some ion channels due to evolutionary modifications. Family with sequence similarity (FAM) proteins, where Piezo1/2 were recognized from, are also a large collection of ~200 miscellaneous proteins (including non-transmembrane ones) with unknown functions. Coiled-coil domain-containing (CCDC) proteins are potential complex-forming proteins with ~150 members, one of which has been found to be the pore-forming subunit of the mitoK_ATP_ channel (CCDC51/MITOK). The 70 members of the leucine-rich repeat-containing (LRRC) proteins, including VRAC components LRRC8A-E and big-conductance K^+^ (BK) channel auxiliary subunits LRRC52/55/38/26 [219], represent one more class of channel candidates or regulators.

**Figure 2:**
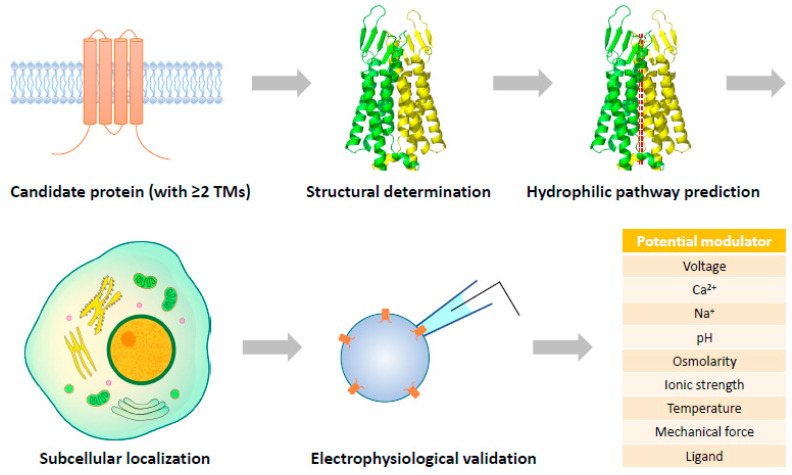
Strategy of structure-guided discovery of unrecognized ion channels. A pore-forming channel protein is expected to have at least two TMs and a hydrophilic ion-conducting pathway that can be detected in either closed or open states.

Identification of novel ion channels may not be essentially limited to proteins of unknown function. Even when a cellular function has been assigned to a transmembrane protein, one cannot simply rule out the possibility that it can be assembled into an ion channel complex. Although not accomplished, the F-type ATP synthase in the inner mitochondrial membrane has been considered the leading candidate for mitochondrial permeability transition pore (mPTP) with structural evidence [220,221]. It can be expected that along with the rapid progress of structural determination of transmembrane proteins, a certain number of novel pore-forming complexes will be identified and subjected to electrophysiological validation (Figure 2). This is a completely reverse way compared to the traditional route of ion channel discovery, and probably the most rational strategy to recognize new ion channels in the cryo-EM era.

For structural biologists, it is routine work to characterize potential hydrophilic pathways within a protein complex even when it is in a closed state. If an ion-conducting pore is structurally predicted, the most challenging task will be assigned to electrophysiologists: to find out how to activate the structurally specified ion channel candidates. Various factors, such as voltage, Ca^2+^, Na^+^, pH, osmolarity, ionic strength, temperature, and mechanical force, need to be tested. If none of these works, the possibility of the candidate being a ligand-gated channel could be considered, which may require high-throughput screening of metabolites to catch the activating molecules. Single-cell expression analyses and subcellular localization of the candidates will also provide useful information to help uncover their activation mechanisms by shortlisting physiological factors with potential actions on the candidates.

## 7. Conclusions

The characterization of electrical currents generated by specific ion channels on cell membranes with the patch clamp technique is often interfered with by electrical noise from the equipment, leak conductance from unstable micropipette–membrane seal, and background currents of the cells. In reconstituted proteoliposomes and artificial lipid bilayers with purified proteins, electrophysiological recordings are frequently associated with membrane leak currents and contamination of endogenous channel proteins from the expression system. These technical issues and the selective use of data contributed to most cases of misidentified channel proteins.

Cryo-EM has been demonstrated to be a powerful tool to validate the pore-forming ability of claimed channel proteins. To assemble an ion-permeable transmembrane structure, proteins should be capable of creating a hydrophilic pathway for ions to pass through the overall hydrophobic environment. Even when an ion channel structure is in the closed state, this hydrophilic pathway should be still identifiable, with the potential to expand into the open state by small conformational changes.

The large number of understudied transmembrane proteins is the most important source of unrecognized ion channels. With the fast progress of cryo-EM structural determination and the assistance of artificial intelligence, structural data will not only be used to interpret the molecular basis of protein functions already known, but also to predict unidentified functions of proteins de novo. When integrated with conventional approaches, this structure-guided functional prediction strategy will be very useful for identifying more ion channels with unusual properties.

## Data Availability

All structural data used to prepare figures in this paper are from published studies and available from RCSB Protein Data Bank.

## Supplementary Materials

The following supporting information can be downloaded at: https://www.mdpi.com/article/10.3390/cells12141870/s1, Figure S1. Cryo-EM structures of TMEM175; Figure S2: Cryo-EM structure of Otop 1; Figure S3: Cryo-EM structures of human proton activated chloride (PAC) channel; Figure S4: Cryo-EM structures of volume regulated anion channel (VRAC); Figure S5: Cryo-EM structures of calcium homeostasis modulators (CALHMS); Figure S6: Structures of OSCA 1.2 and TMEM 16; Figure S7: Touch-sensitive plants and cryo-EM structure of Flycatcher 1; Figure S8: Cryo-EM structure of the ribosome-TMCO1 translocon complex; Figure S9: Cryo-EM structure of TMEM120A; Figure S10: Cryo-EM structure of human TMEM87A; Figure S11: Cryo-EM structures of mouse TTYH2; Figure S12: Structures of SARS-CoV-2 viroporins; Figure S13: Structural comparison of OSCA and TMEM63 proteins; Figure S14: AlphaFold-predicted structures of TMEM150A/B/C; Figure S15: Structural comparison of the components of plasma membrane KATP and mitoKATP; Figure S16: AlphaFold-predicted structure of human SLCO2A1; Figure S17: Function and structure of MLKLprotein; Figure S18: Hypothesis of the channel-forming process ofCLIC1.
