# Supplementary material for: To Be or Not to Be an Ion Channel: Cryo-EM Structures Have a Say
Source: Cells. 2023 Jul 17;12(14):1870. doi: 10.3390/cells12141870 (PMC10378246; doi:10.3390/cells12141870)
Supplement: Supplementary file 1 [file cells-12-01870-s001.zip › cells-2439061-supplementary.pdf]

Figure S1

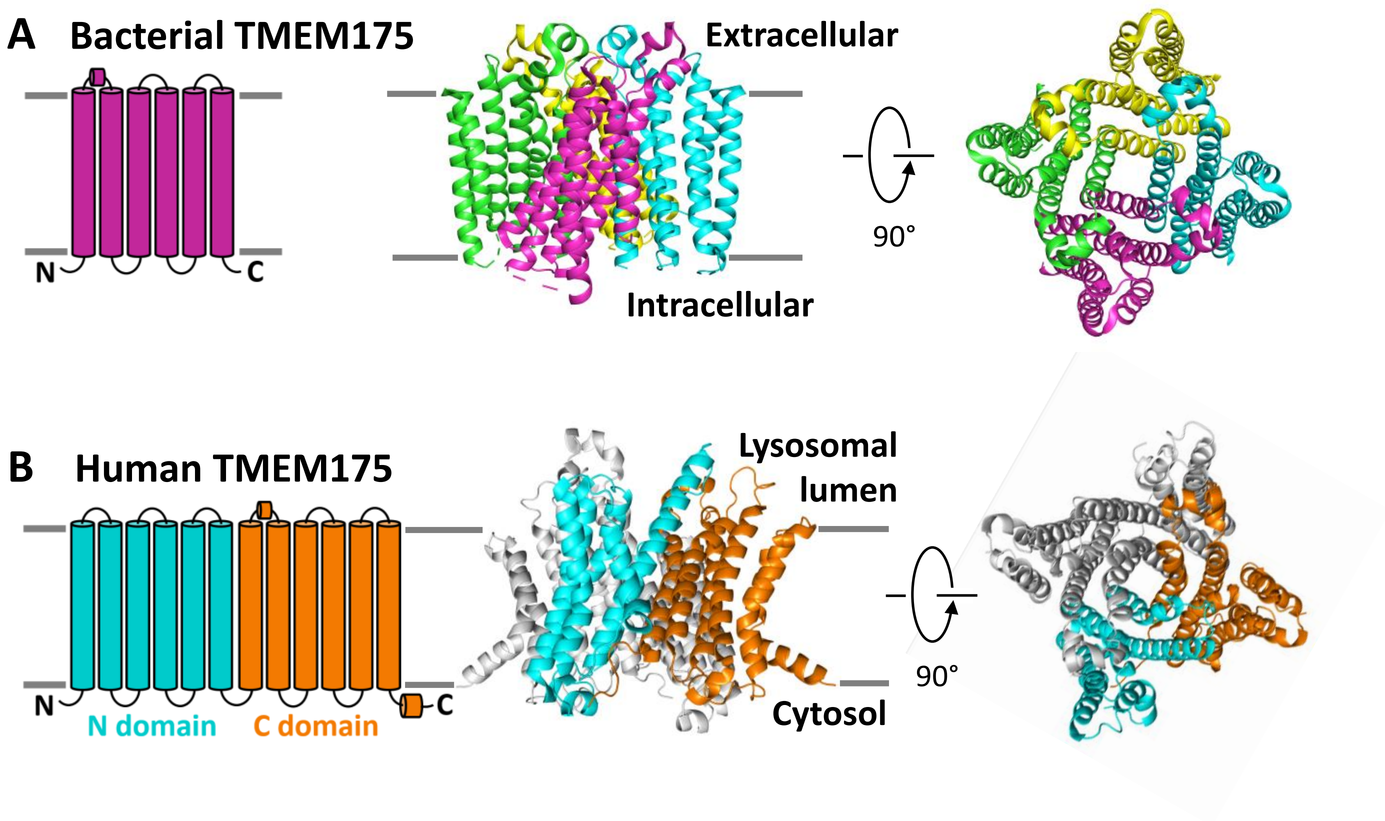

Figure S1. Cryo-EM structures of TMEM175. (A) Schematic topology and cryo-EM structure of bacterial (*Chamaesiphon minutus*) TMEM175 (PDB: 5VRE). (B) Structurally similar N and C domains of human TMEM175 (PDB: 6W8P).

Figure S2

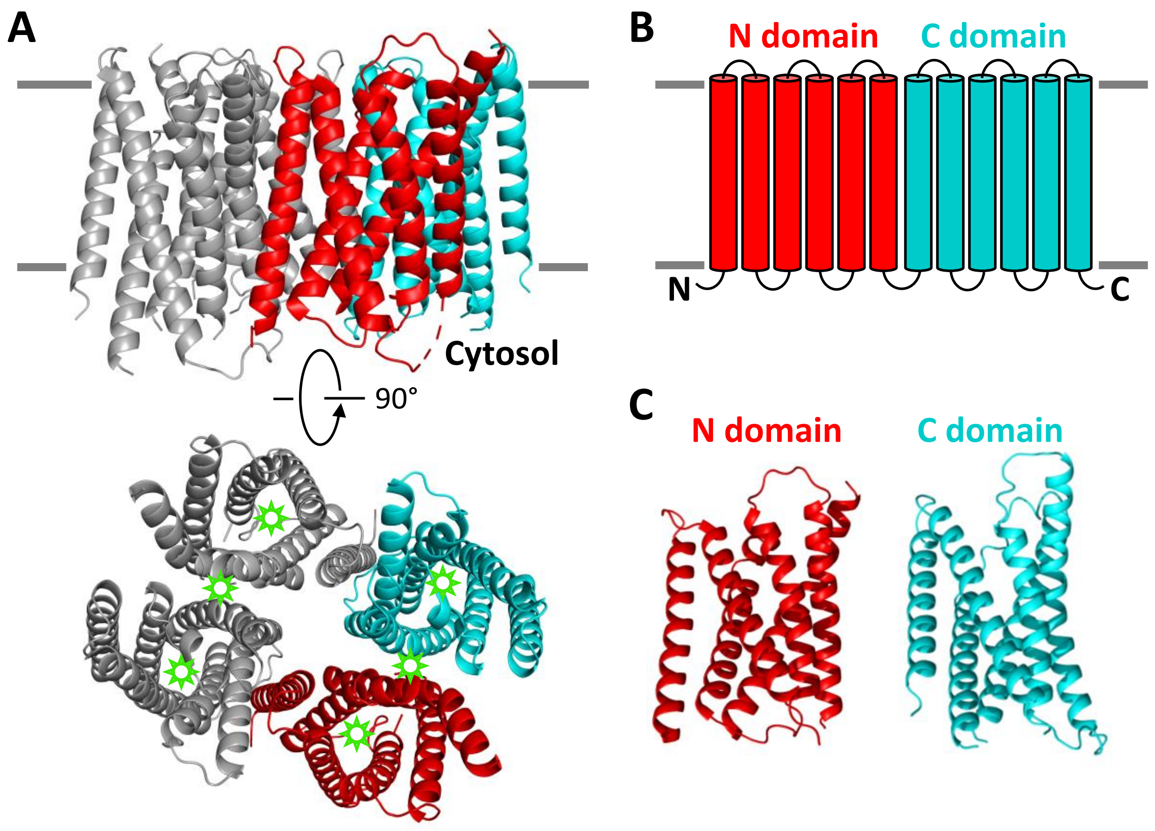

Figure S2. Cryo-EM structure of Otop1. (A) Cryo-EM structure of zebrafish Otop1 (PDB: 6NF4). One protomer is colored red and cyan which the other is colored grey. The green hollow star symbols indicate positions of putative pores. (B) Schematic topology and (C) structural comparison of the N and C domains of Otop1.

Figure S3

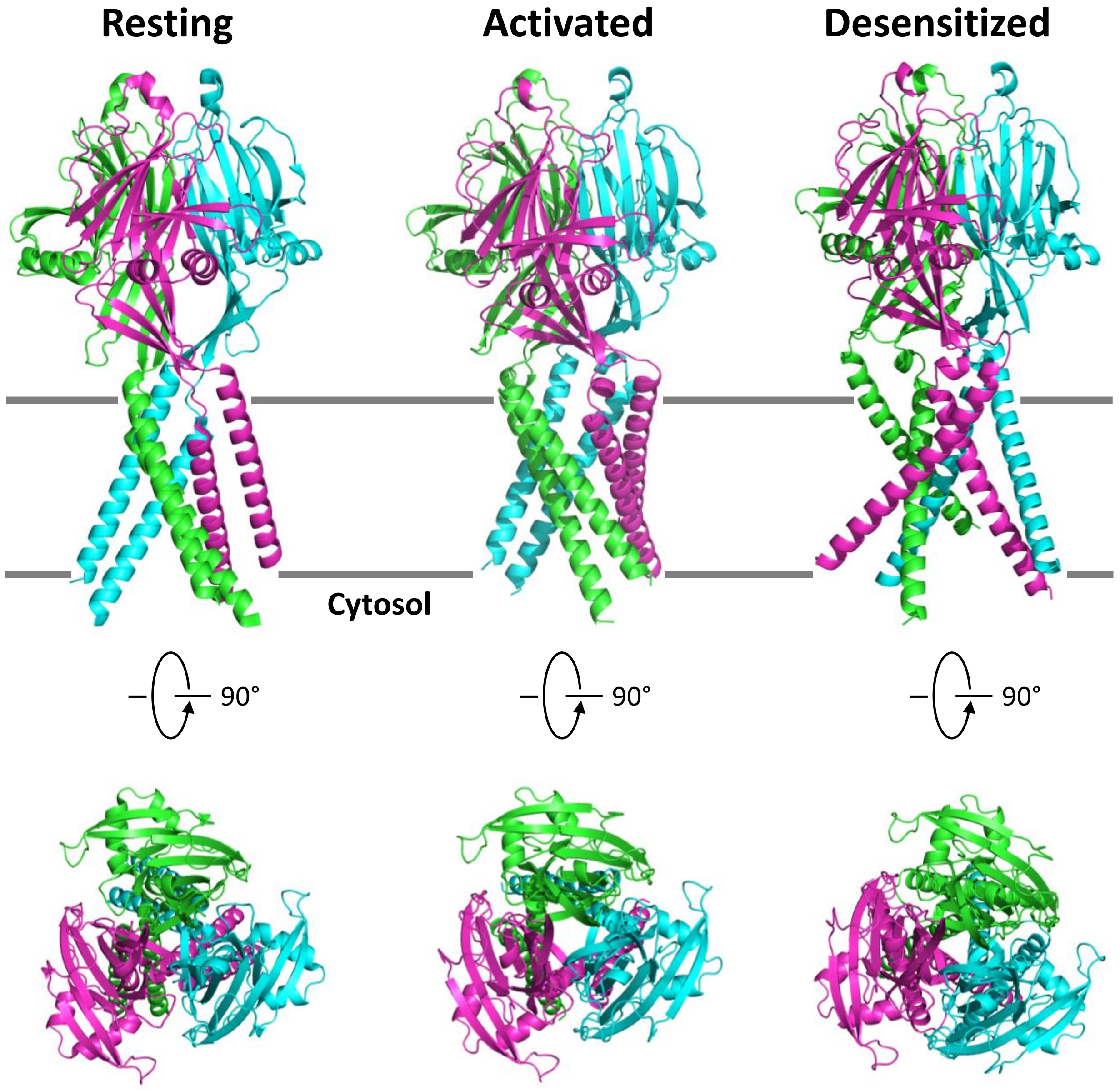

Figure S3. Cryo-EM structures of human proton-activated chloride (PAC) channel. Structures representing the resting (pH 7.5; PDB: 7SQG), activated (pH 4.5; PDB: 7SQF) and desensitized (pH 4.5; PDB: 7SQH) states are shown.

Figure S4

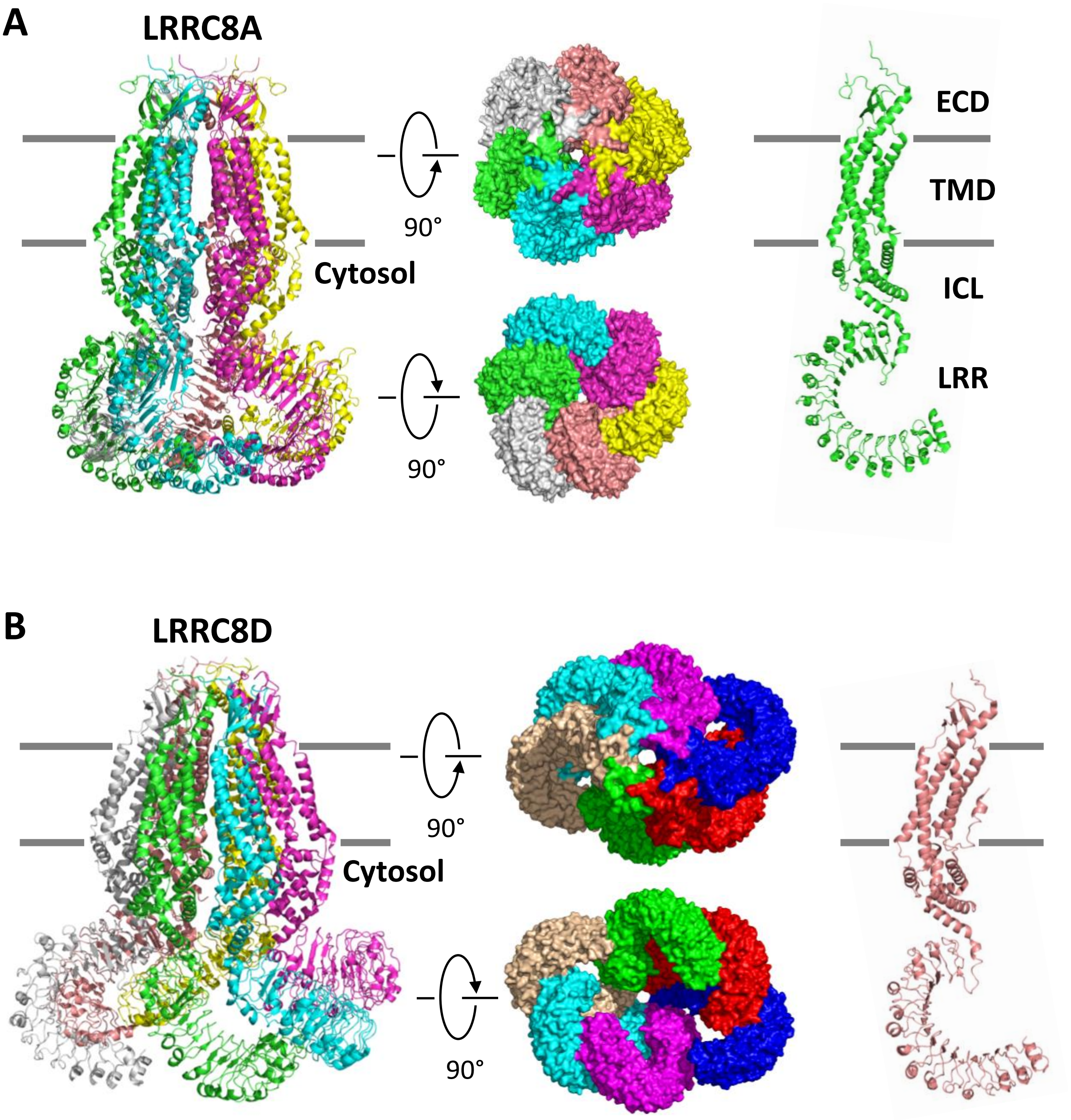

Figure S4. Cryo-EM structures of volume-regulated anion channel (VRAC). (A) Homohexameric and monomeric structures of mouse LRRC8A (PDB: 6G9L). ECD: extracellular domain; TMD: transmembrane domain; ICL: intracellular linker; LRR: leucin-rich repeats. (B) Homohexameric and monomeric structures of human LRRC8D (PDB: 6M04).

Figure S5

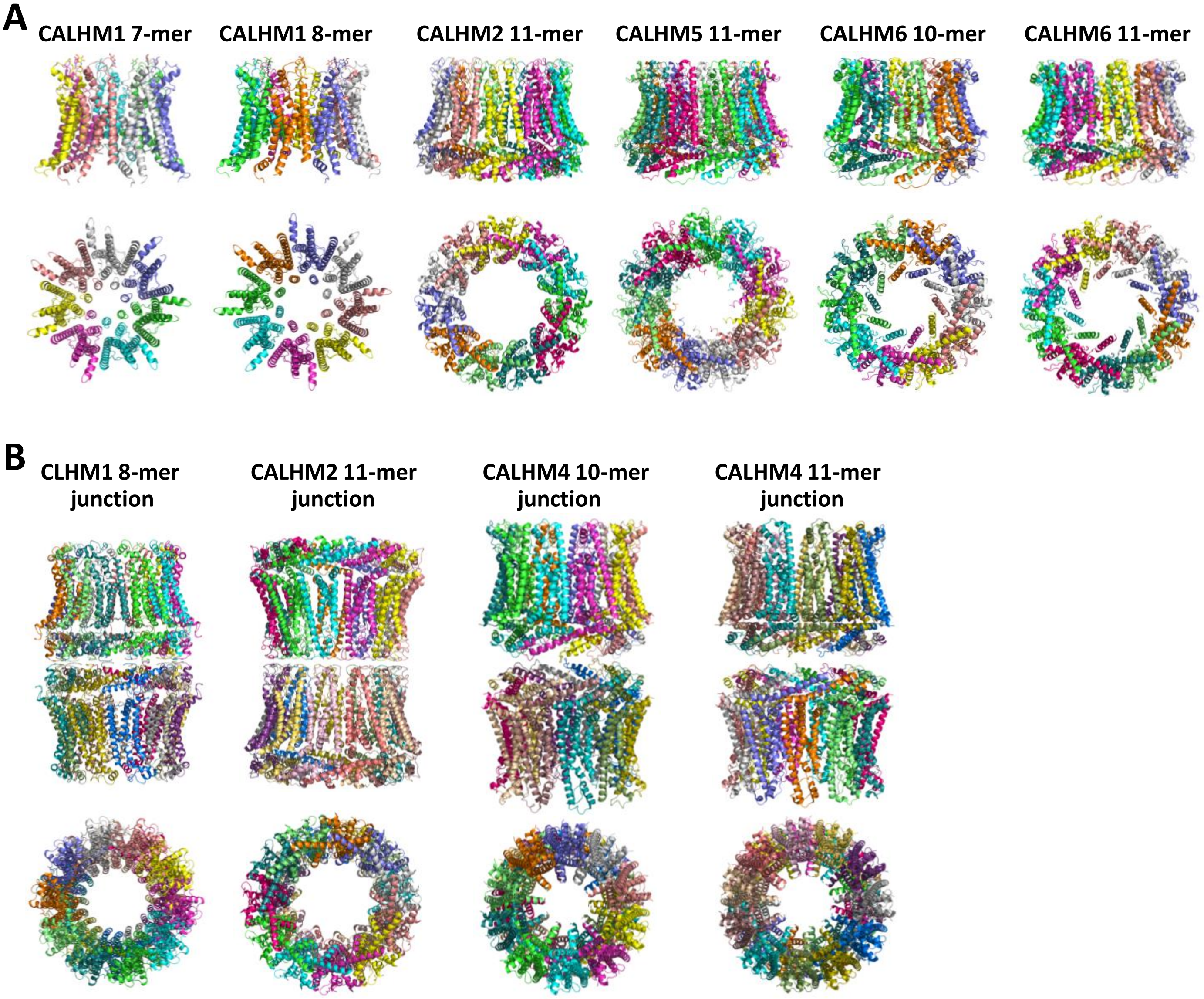

Figure S5. Cryo-EM structures of calcium homeostasis modulators (CALHMs). (A) Homo-oligomers of zebrafish CALHM1 (PDB: 7DSD and 7DSC), human CALHM2 (6VAK), human CALHM5 (7D61) and human CALHM6 (6YTV and 6YTX). (B) Homomeric junctions of *C. elegans* CLHM1 (6LOM), human CALHM2 (6VAI) and human CALHM4 (6YTO and 6YTQ).

Figure S6

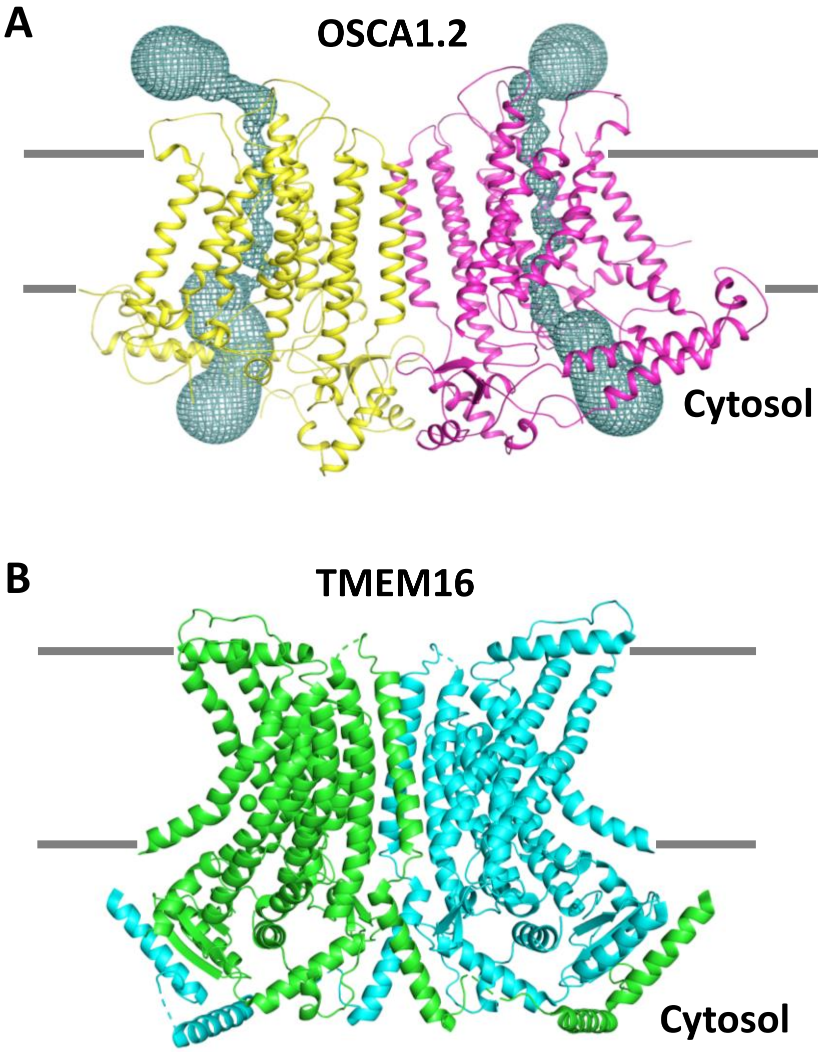

Figure S6. Structures of OSCA1.2 and TMEM16. (A) Cryo-EM structure of rice (*Oryza sativa*) OSCA1.2 with putative pores (PDB: 6OCE). (B) Crystal structure of fungus (*Fusarium vanettenii*) TMEM16 (PDB: 4WIS).

Figure S7

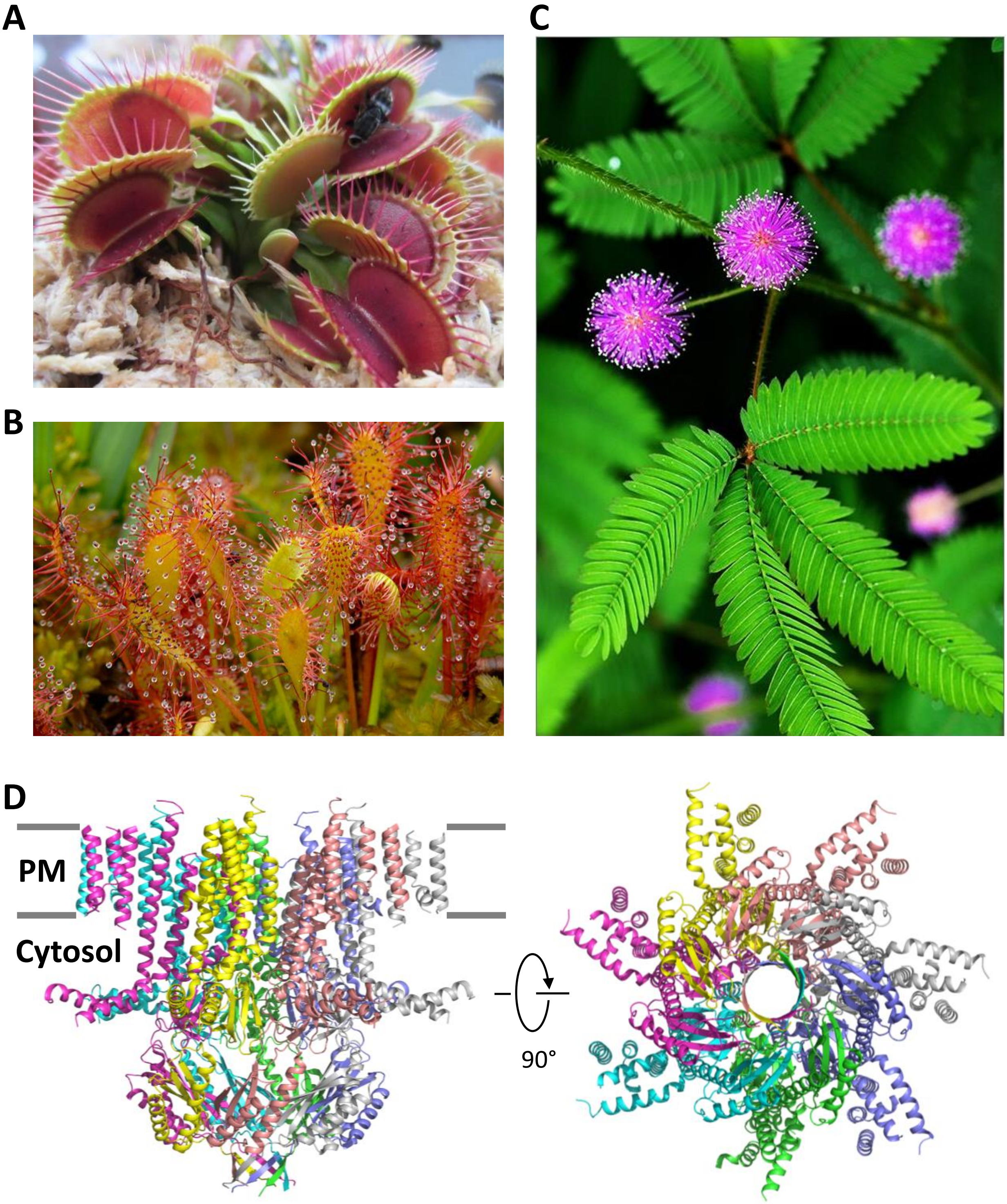

Figure S7. Touch-sensitive plants and cryo-EM structure of Flycatcher1. (A) Venus flytrap (*Dionaea muscipula*). (B) Cape sundew (*Drosera capensis*). (C) *Mimosa pudica*. (D) Cryo-EM structure of Venus flytrap Flycatcher1 (PDB: 7N5D).

Figure S8

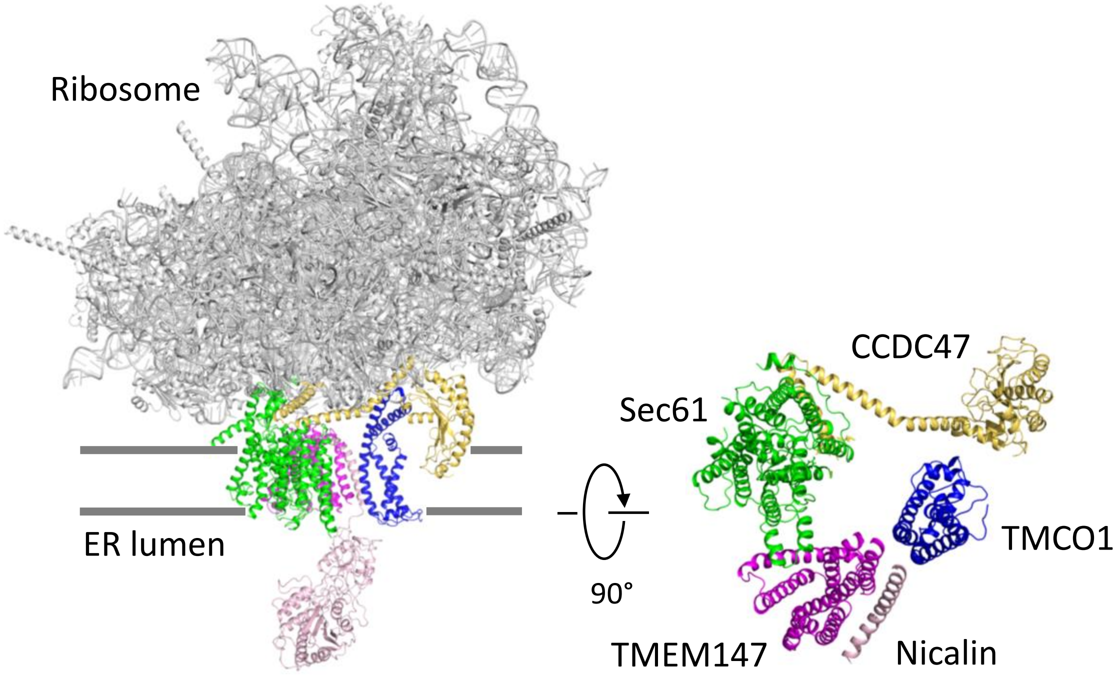

Figure S8. Cryo-EM structure of the ribosome-TMCO1 translocon complex. Components of the translocon are colored (PDB: 6W6L).

Figure S9

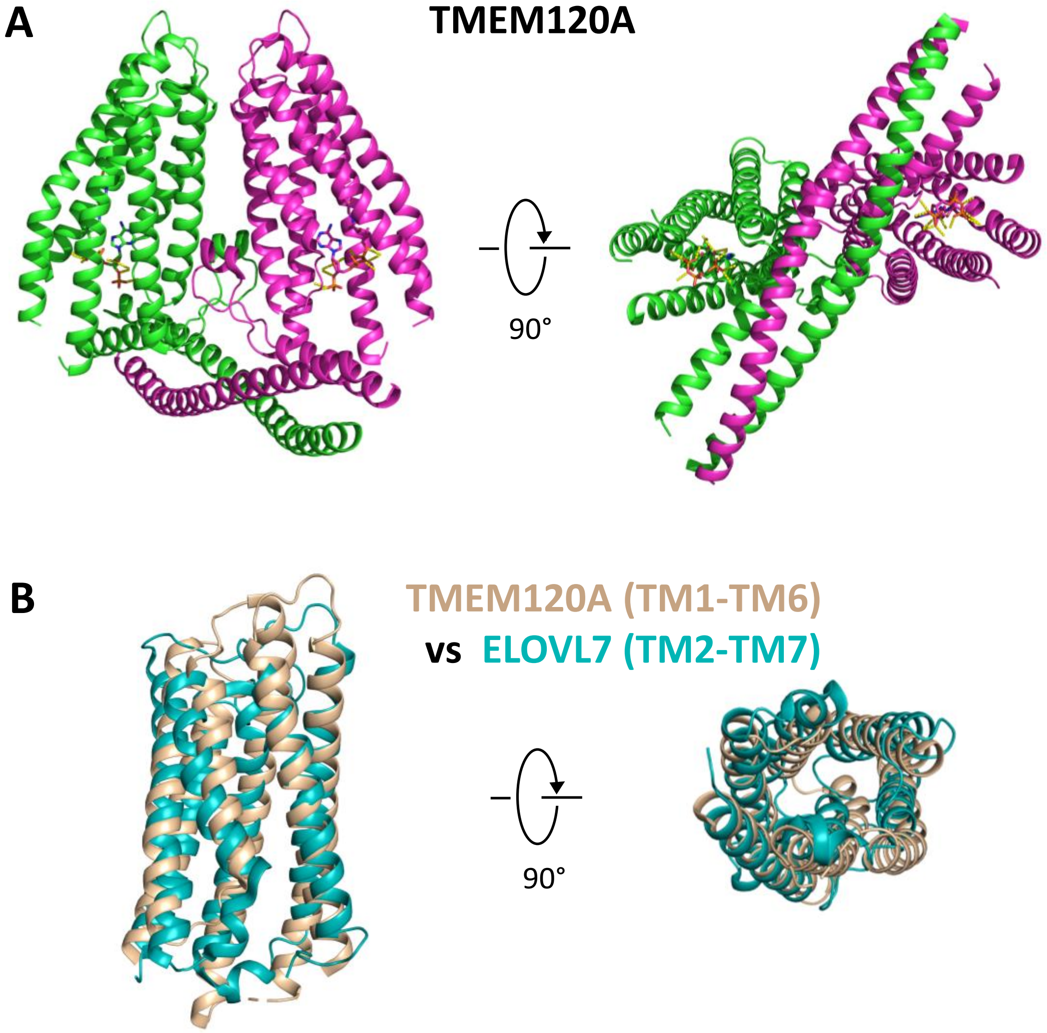

Figure S9. Cryo-EM structure of TMEM120A. (A) Dimeric structure of human TMEM120A (PDB: 7N7P). Coenzyme A molecules bound in TMEM120A are shown in sticks. (B) Structural comparison of the transmembrane helices of TMEM120A and human fatty acid elongase 7 (ELOVL7; PDB: 6Y7F).

Figure S10

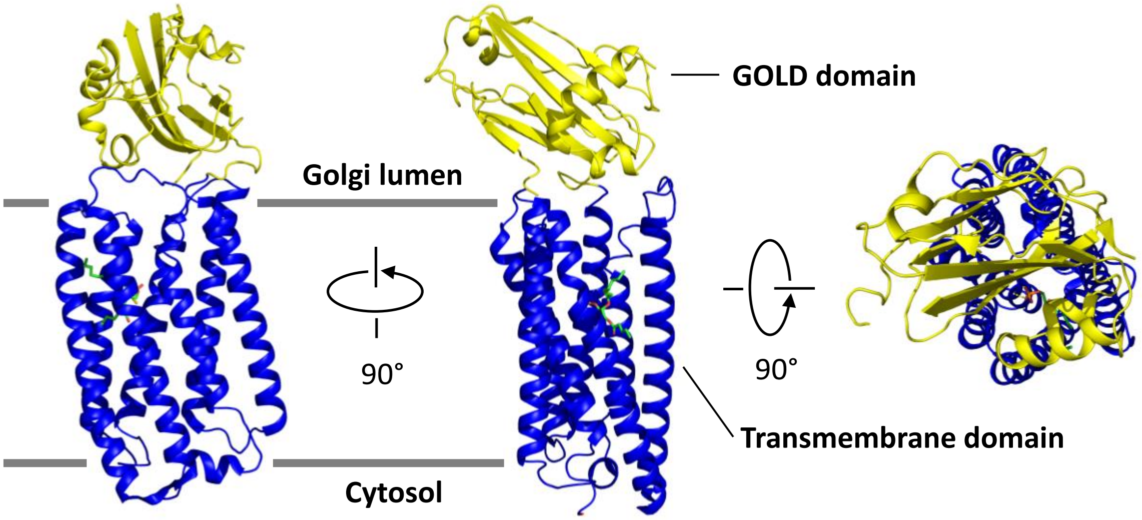

Figure S10. Cryo-EM structure of human TMEM87A. Golgi-dynamics (GOLD) and transmembrane domains are colored yellow and blue, respectively. Phospholipids are shown in sticks. (PDB: 8CTJ)

Figure S11

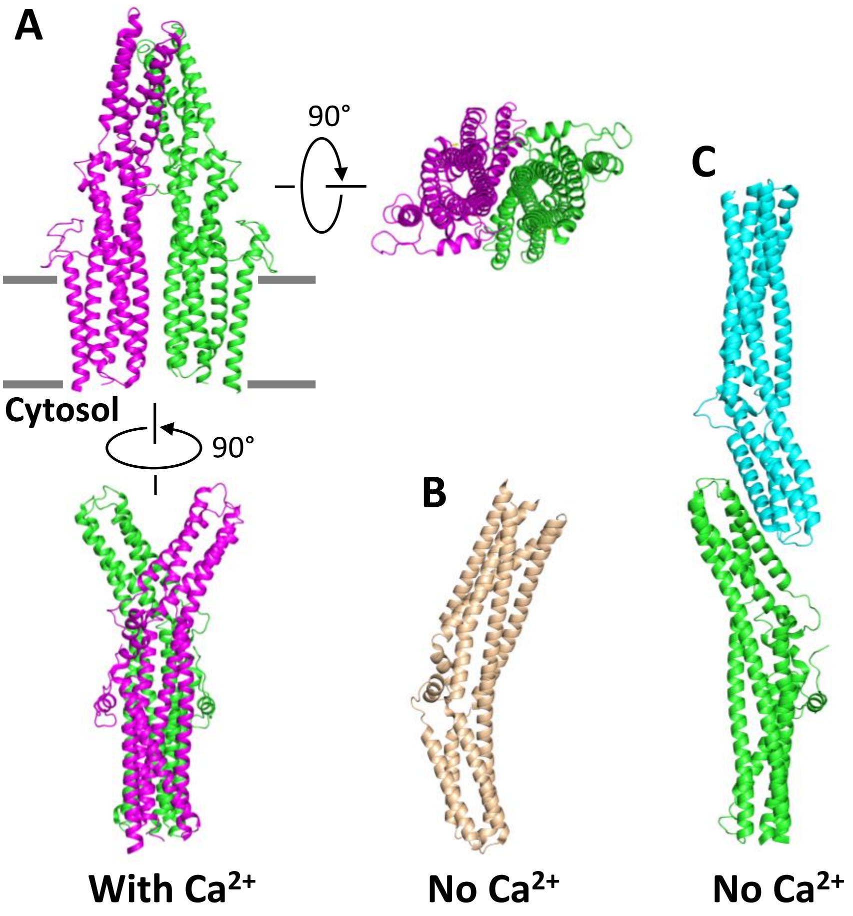

Figure S11. Cryo-EM structures of mouse TTYH2. (A) Dimeric structure of TTYH2 in the presence of Ca^{2+} (PDB: 7RTT). (B) Monomeric and (C) dimeric structures of TTYH2 without Ca^{2+} (PDB: 7RTV and 7RTU).

Figure S12

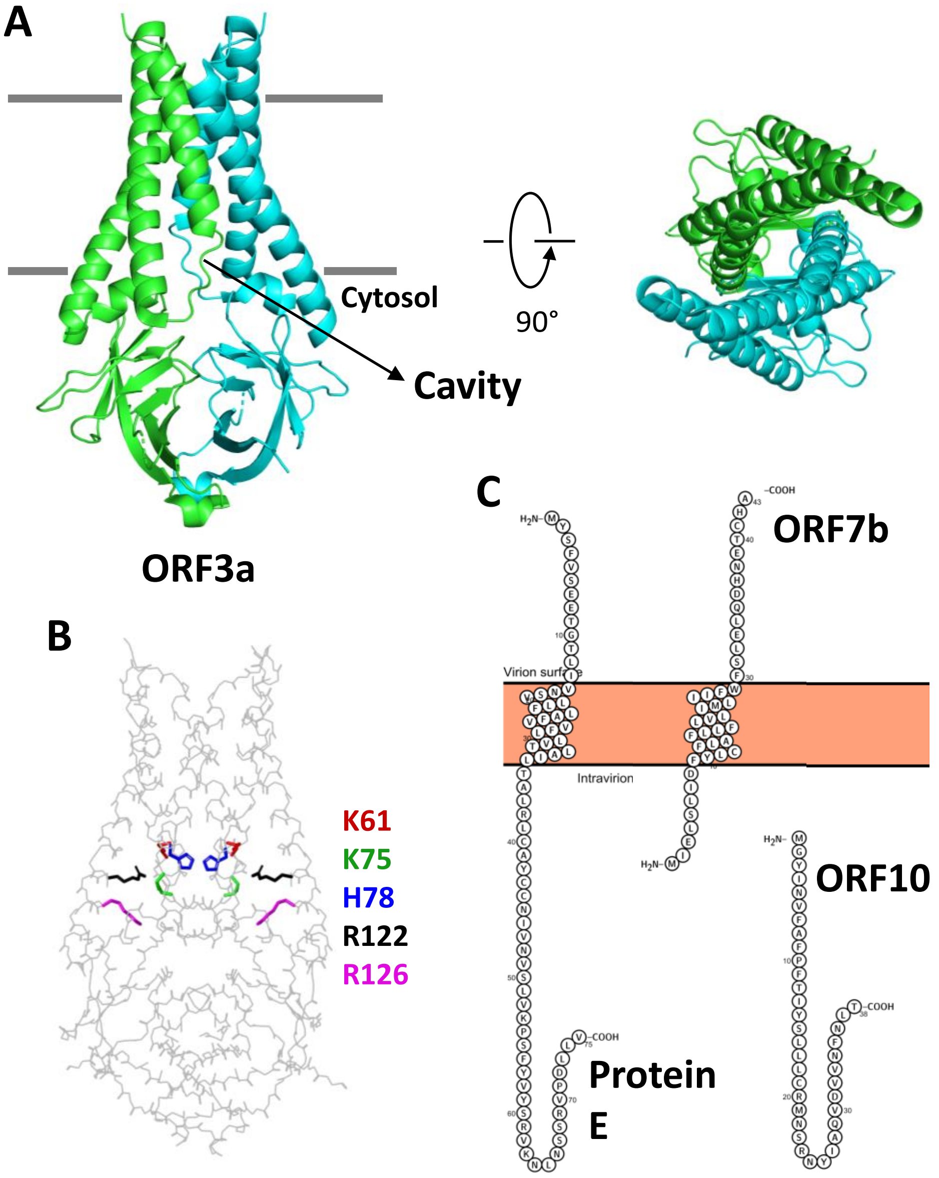

Figure S12. Structures of SARS-COV-2 viroporins. (A) Cryo-EM structure of SARS-COV-2 ORF3a (PDB: 7KJR). (B) Positively charged residues in the aqueous cavity of SARS-COV-2 ORF3a. (C) Transmembrane topology of SARS-COV-2 protein E, ORF7b and ORF10 predicted by Protter (https://wlab.ethz.ch/protter/).

Figure S13

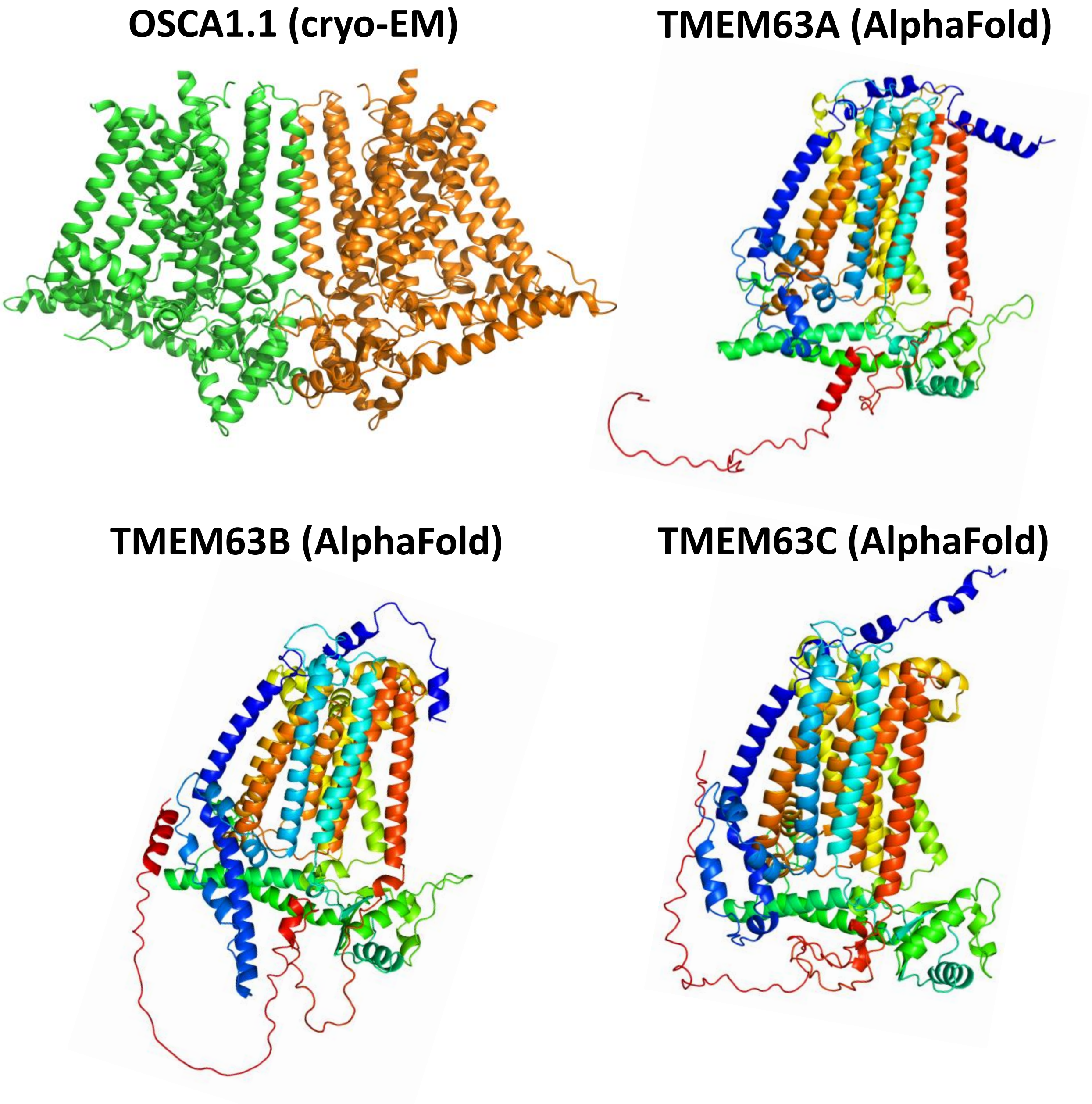

Figure S13. Structural comparison of OSCA and TMEM63 proteins. (A) Cryo-EM structure of *Arabidopsis thaliana* OSCA1.1 (PDB: 6JPF). (B-D) AlphaFold-predicted structures of human TMEM63A/B/C.

Figure S14

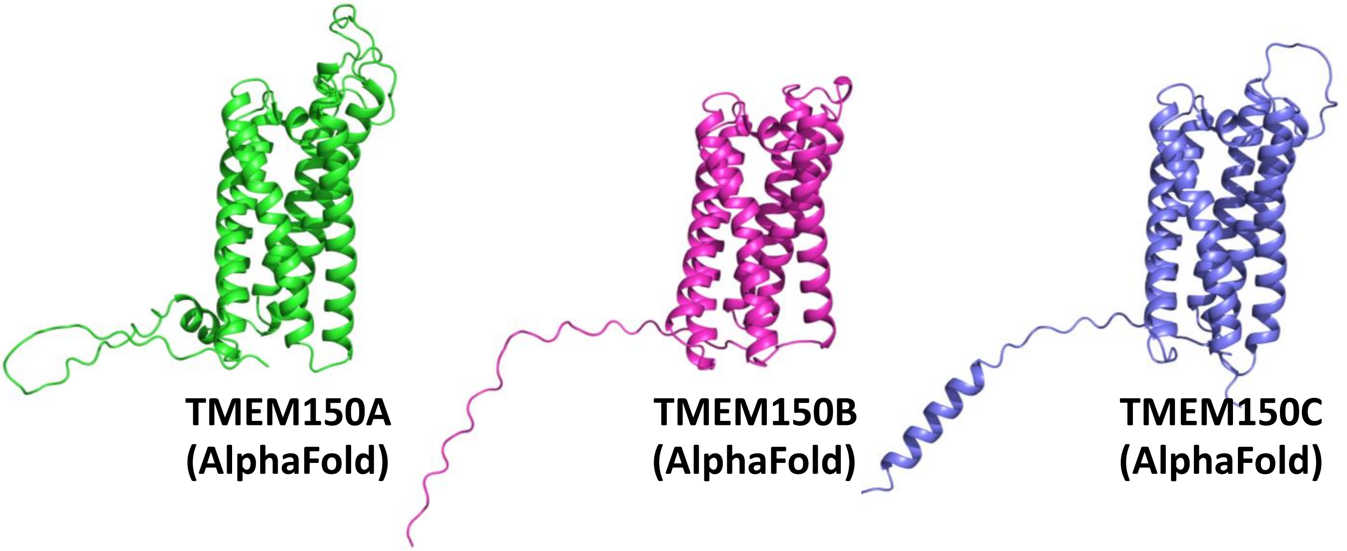

Figure S14. AlphaFold-predicted structures of TMEM150A/B/C.

Figure S15

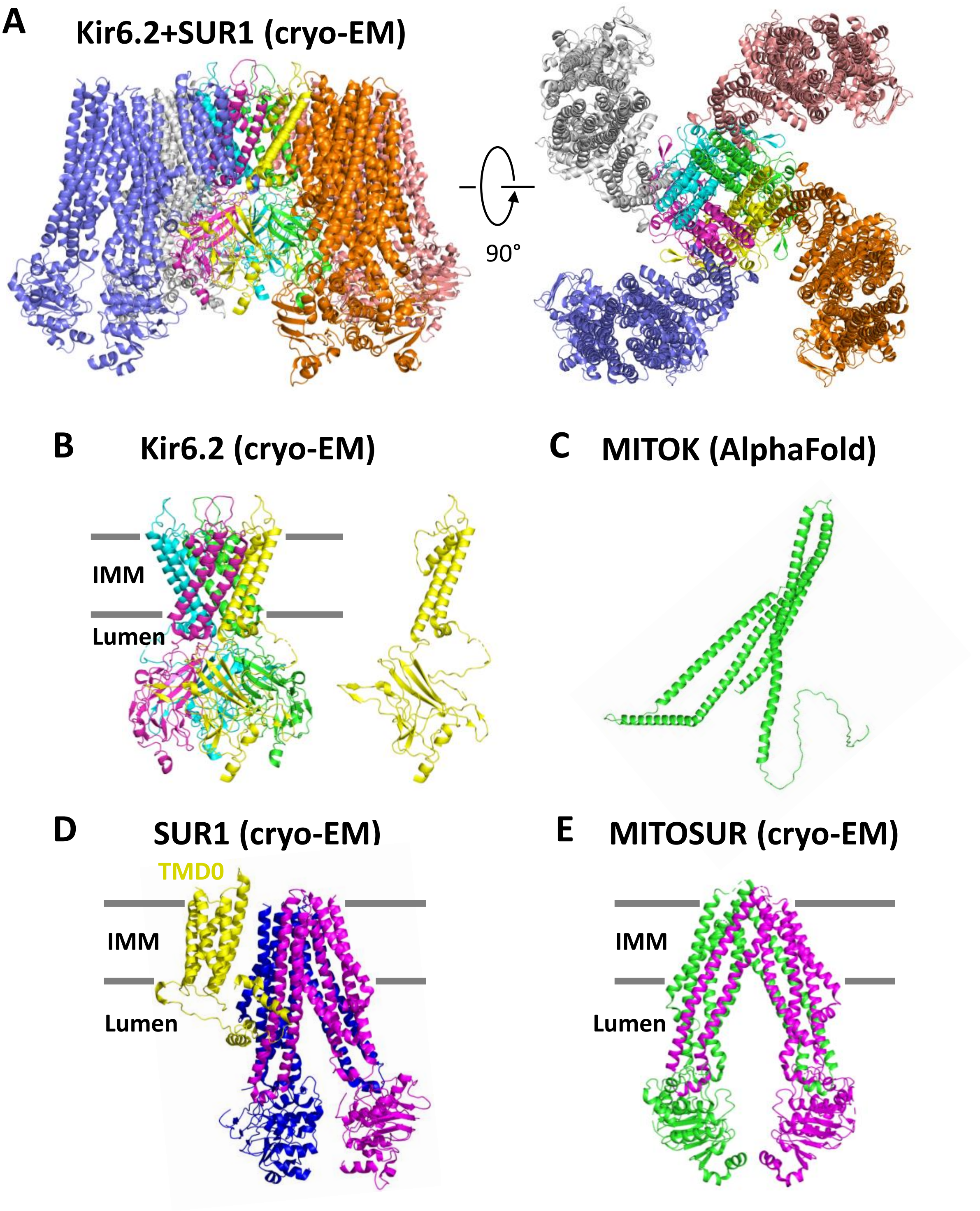

Figure S15. Structural comparison of the components of plasma membrane K_{ATP} and mitoK_{ATP}. (A) Cryo-EM structure of K_{ATP} (mouse K_{ir}6.2 and golden hamster SUR1; PDB: 5WUA). (B) Tetrameric and monomeric structures of mouse K_{ir}6.2. IMM: inner mitochondrial membrane. (C) AlphaFold-predicted structure of human MITOK (CCDC51). (D) Monomeric structure of golden hamster SUR1. TMD0: transmembrane domain 0. (E) Dimeric cryo-EM structure of human MITOSUR (ABCB8; PDB: 7EHL).

Figure S16

SLCO2A1 (AlphaFold)

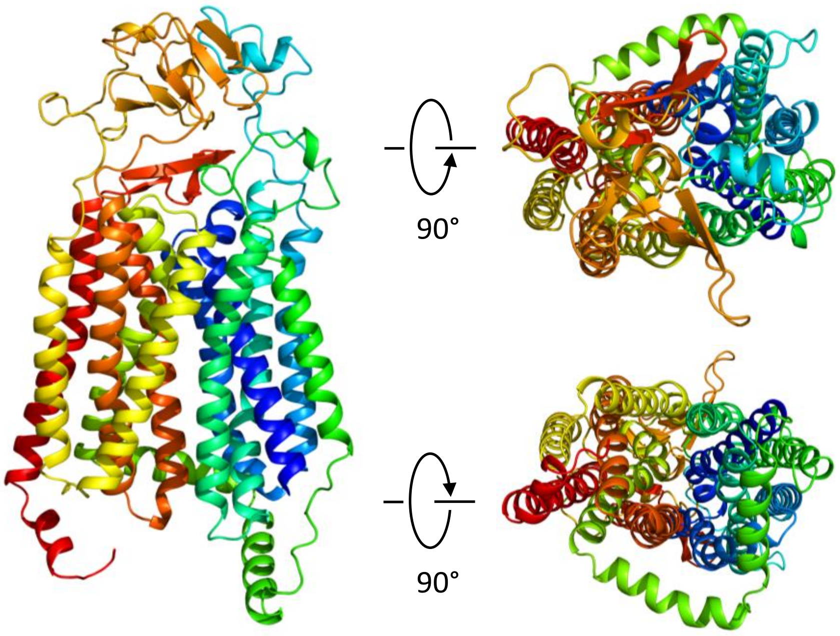

Figure S16. AlphaFold-predicted structure of human SLCO2A1.

Figure S17

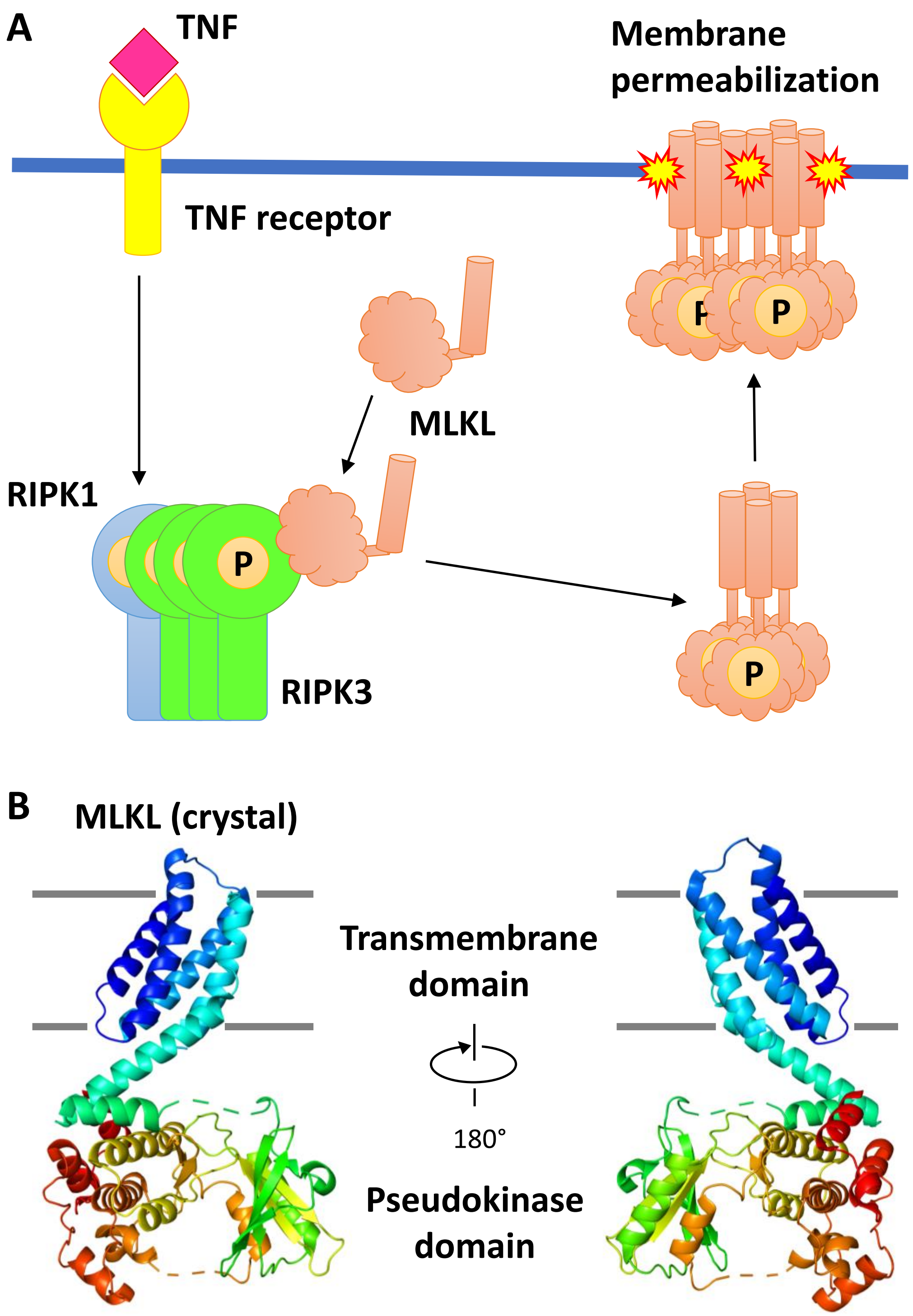

Figure S17. Function and structure of MLKL protein. (A) Signal transduction in necroptosis. (B) Crystal structure of unphosphorylated mouse MLKL protein (PDB: 4BTF).

Figure S18

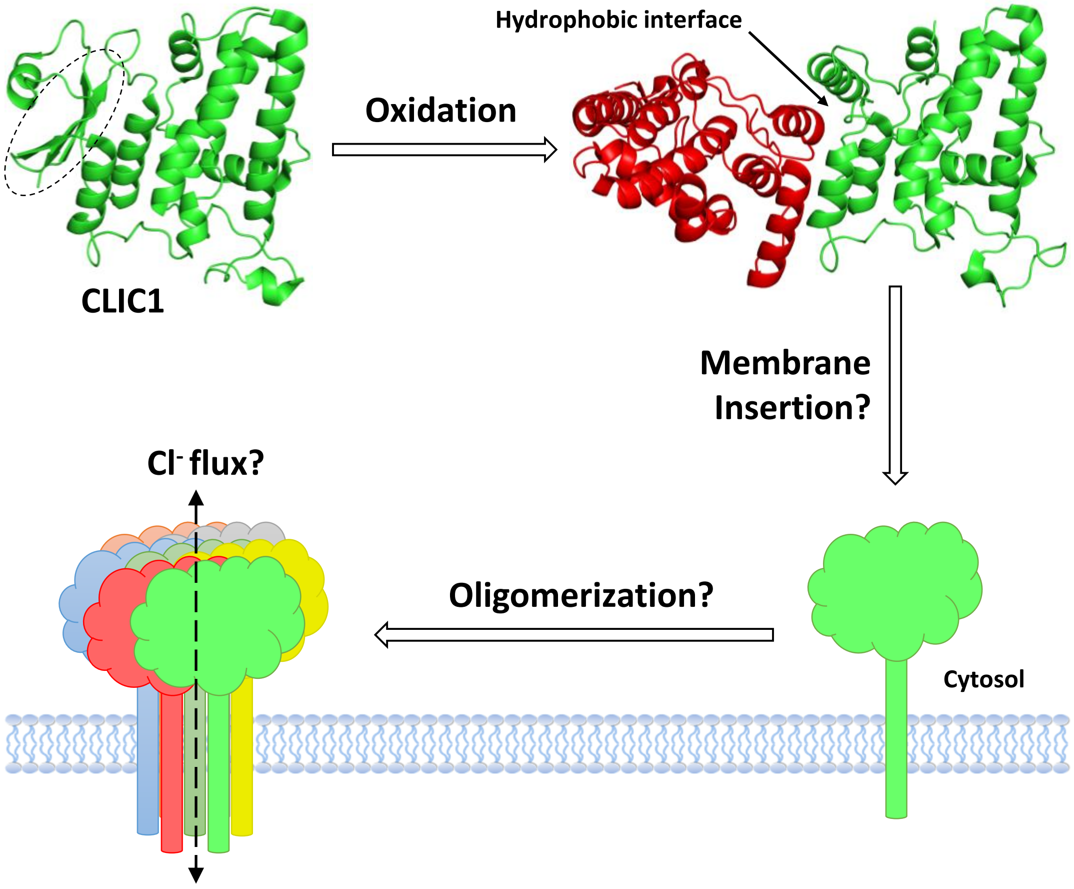

Figure S18. Hypothesis of the channel-forming process of CLIC1. Crystal structures of human CLIC1 at reducing (monomer; PDB: 1K0M) and oxidizing (dimer; PDB: 1RK4) conditions are shown. The dashed oval indicates the N-terminal four-stranded β sheet that transformed into helical structure upon oxidation, where a hydrophobic surface is exposed to facilitate the dimeric interaction.
